# The unique biology of catch muscles: insights into structure, function, and robotics innovations

**DOI:** 10.3389/fbioe.2025.1478626

**Published:** 2025-04-16

**Authors:** Ilya Vyatchin, Vyacheslav Dyachuk

**Affiliations:** Laboratory of Cell Biophysics, A.V. Zhirmunsky National Scientific Center of Marine Biology, Russian Academy of Sciences, Vladivostok, Russia

**Keywords:** catch state, thick and thin filaments, myorod, twitchin, myogenesis, development, single cell transcriptomic analysis, robotic mechanisms

## Abstract

The Review covers the current state of functions, neurotransmitter innervation, the structure, and development of the contractile apparatus of unique group of catch muscles inherent only to bivalves. In contrast to conventional muscles, during contraction and relaxation, catch muscles possess a unique ability to enter the contraction holding state, referred to as catch state. The latter consists in energy-efficient maintenance of long-lasting tension developed by the muscle without consuming ATP-derived energy and regulated by serotonin and acetylcholine. Despite the molecular mechanism of catch state phenomenon still remains unclear, the combination of experimental data and the resulting assumptions allow one to design new energy-efficient and chemically-driven artificial muscles. The analysis of the structure and function of the catch muscles in this work opens the way to a conceptually new strategy for energy-efficient biomimetic robotics, including underwater robotics.

## 1 Introduction

Mechanisms designed by nature are in many aspects far superior to those designed by humans. Ordinary and familiar to us muscle tissues - are characterised by very high developed force (relative to mass), elasticity and softness, speed and frequency of contraction, extraordinary energy efficiency, environmental friendliness. But the most important thing is that muscle tissues, by their cellular nature, have reparative potential and, therefore, in theory, unlimited resource and service life. All the advantages of biological muscles mentioned here can be fairly attributed to the disadvantages of mechanical drives. Of course, taking into account all the advantages of natural muscle tissues, the creation of artificial bio-muscles, or bio-drives based on the work of motor proteins, seems to be an extremely interesting prospect.

There are many unresolved challenges on the way to realising this vision. The main one is the lack of an exhaustive understanding of how muscles work and how they realise certain properties and advantages. On the one hand, we have really well studied the striated (skeletal) muscles of vertebrates. And, of course, these tissues could be the primary basis for the creation of bio-actuators, but they are much inferior to smooth muscle tissues in a number of characteristics. For example, smooth muscles are characterised by extremely high strength and endurance. Moreover, they have a monomeric (cellular) organisation that is incredibly successful for scaling. The most striking example of smooth muscles that combines all these properties is the catch muscles – smooth adductors and retractors of bivalves and it is these muscles that will be the focus of most of this review. In addition to the advantages mentioned above, the catch muscles of molluscs have a unique advantage that makes them different from other biological actuators. These muscles are capable of the catch state, in which the muscle does not expend energy to maintain the force it has generated and does not show signs of fatigue. At the same time, the rate of energy expenditure and fatigue accumulation of skeletal muscles, for example, is very high (the reader can easily test this by freezing in a static posture, such as raising the arm upwards). Strangely enough, this ability of the catch muscle to freeze brings it closer to classic human-made engines and mechanisms, which use almost no energy at rest.

Due to their unique ability, the catch muscles of bivalves seem to be the most promising basis for the creation of synthetic bio-actuators. However, their molecular structure, regulation of function and development are not yet fully understood enough to directly reproduce their mechanisms in bio-robotics. In order to speed up the study of these mechanisms and their direct application, we have tried to bring together the disparate information on the catch muscle. We are talking about the molecular organisation of the contractile apparatus of the catch muscle, the properties and possible functions of the proteins that make it up, and the development and innervation of the muscle. We think that this information will be useful in the future for the creation of hybrid muscles, in which proteins and structures of different origin can be used, the set of which can be arbitrarily determined by the tasks to be solved. In this paper we have tried to outline the trajectory and problems of biotechnology development in this direction.

### 1.1 Tissue structure and cell anatomy

Catch muscles are separately located smooth muscles of bivalves, fixed by one or both ends to the mollusc shell. Accordingly, among these muscles we can distinguish both adductors (muscles that close the mollusc shell in case of danger) and retractors (retracting byssus or leg). Both are distinguished by the need to maintain the tension developed by the muscle for a prolonged period of time (Twarog, 1967a). This necessity has led to the emergence of a special state in the catch muscles, the catch state (Parnas, 1910), in which the force developed by the muscle is maintained with virtually no energy expenditure. No other known muscle is capable of this. As a result, these muscles combine the advantages of smooth muscles with a unique ability that can be extremely useful in robotics. However, in other respects, this muscle also performs outstandingly well, once again confirming our opinion that this is the muscle to start with when creating bio-actuators.

**FIGURE 1:**
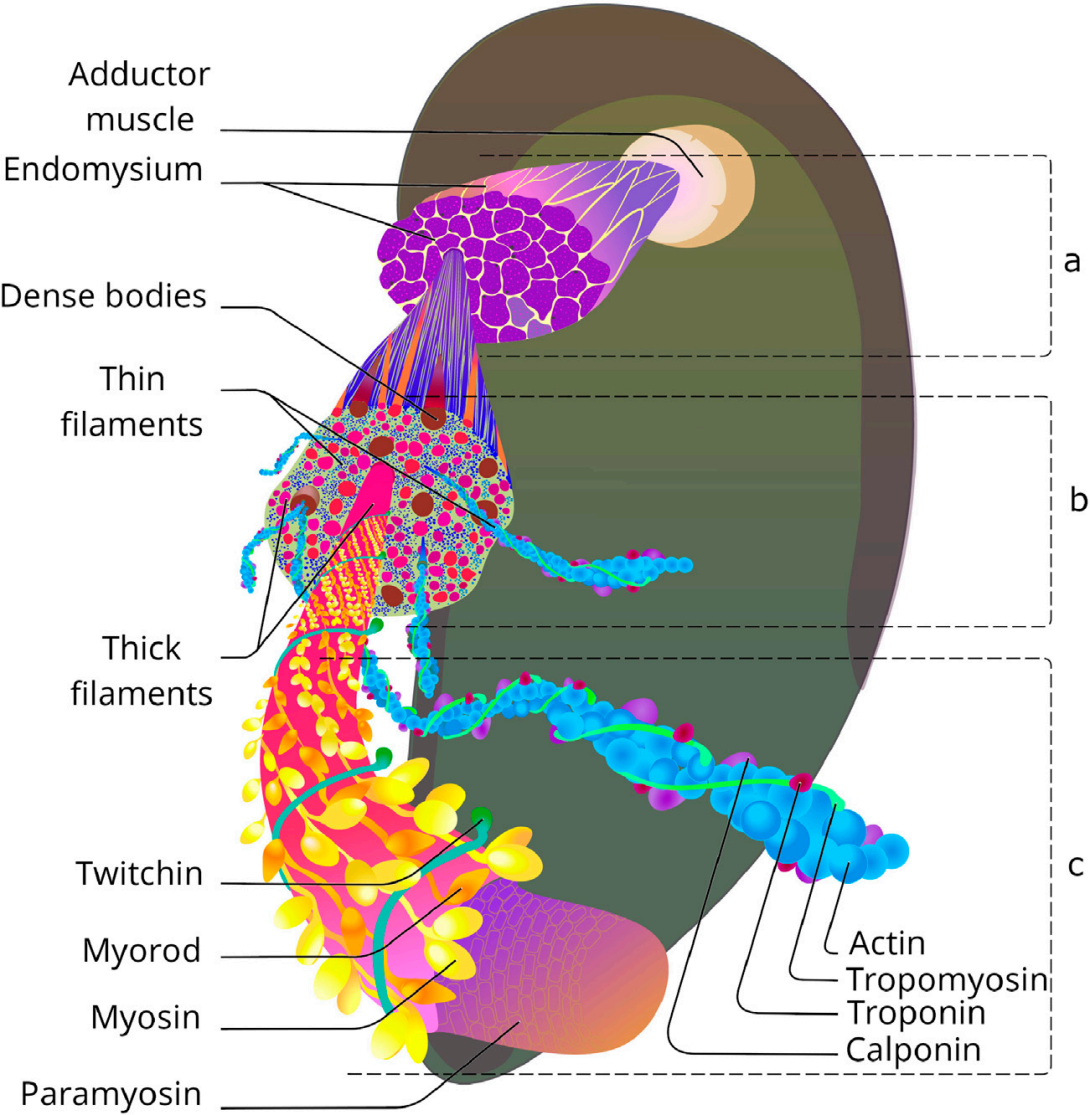
Catch muscle organisation: from tissue to molecular level. **(a)** Group of smooth cells separated with endomysium. Based on (Sobieszek, 1973). **(b)** Transverse section of smooth muscle cell. Based on (Sobieszek, 1973; Bennett and Elliott, 1989; Takahashi et al., 2003). **(c)** Molecular structure of contractile filaments. Based on (Szent-Györgyi et al., 1971; Hooper et al., 2008; Tajima et al., 1983; Lehman et al., 2009).

Firstly, like other smooth muscles, the catch muscle has cellular rather than symplastic structure. The muscle tissue consists of a multitude of spindle-shaped cells connected to each other and to matrix by hemidesmosomes, nexal junctions (15 nm long), and other-type junctions, representing the convergence of the sarcolemma of neighbouring cells, up to several micrometres long (Kennedy et al., 1996). This arrangement of muscle tissue allows for fairly simple production of cells in cell culture, and easy scaling of the size of the tissue produced. The cells themselves are about 1.6 mm long [three times longer than some of the longest vertebrate smooth muscle cells (KURIYAMA et al., 1998)], and 4–20 µm in diameter (twice the size of their vertebrate counterparts). The cells are surrounded by endomysium, a thin layer of connective tissue containing collagen fibres, the volume of which in the tissue does not exceed 20% (Figure 1). Nerve endings and blood vessels approach the cells through the endomysium, haemolymphatic sinuses open into it (Kennedy et al., 1996). Each of the cells has an individual contractile and repair apparatus, which can also be very important when creating artificial muscles of different sizes. Cell nuclei (22 µm in diameter) are located at the periphery, surrounded by mitochondria. The calcium apparatus is represented by the endoplasmic reticulum, centred along the surface of the sarcolemma (Atsumi and Sugi, 1976). Most of the cell volume is occupied by the contractile apparatus, which does not show a strict sarcomeric organisation.

Nevertheless, something similar to sarcomeres can be distinguished - these are areas in which thick filaments are surrounded by thin filaments parallel to them (a ratio of about 1:18). Z-lines, to which thin filaments are attached in striated muscles, are absent; instead, dense bodies with polar structure are located in the thickness of the contractile apparatus and on the sarcolemma. These organelles are 1.8 µm long and about 0.12 µm in diameter, with 60–160 thin filaments attached to each. The dense bodies at the ends of neighbouring cells are particularly numerous and arranged symmetrically, apparently to transmit contractile force through the muscle tissue. Similar to the Z-discs of transverse striated muscles, the dense bodies of the catch muscles confine individual sarcomeric structures that are about 50 μm long. This is almost 20 times longer than the sarcomere of skeletal muscles (Enoka and Duchateau, 2019) and the contractile unit of vertebrate smooth muscles (Herrera et al., 2005).

Structurally, the contractile unit of a muscle consists of two-halves of an opposing dense bodies, with 60–80 thin filaments (10 µm long) directed from each to the centre of the sarcomere, and 3-4 thick filaments (25 µm long) located between them. As can be seen from the description of the ultrastructure of these muscles - although the catch muscles are outstanding in their parametric characteristics, there are no fundamental differences in the organisation of this tissue from other smooth muscles. However, these differences do exist, and they can be found at the molecular level, which prompts us to analyse the structure of this tissue in more depth. As mentioned above, the contractile apparatus of the catch muscle has the same basic structures as all muscles known to science - thin and thick filaments, and obviously functions according to the theory of sliding filaments. However, when examined in detail, it turns out that behind the external similarities there are profound differences. Let us consider the contractile filaments of this muscle in more detail.

#### 1.1.1 Thick filaments

**FIGURE 2:**
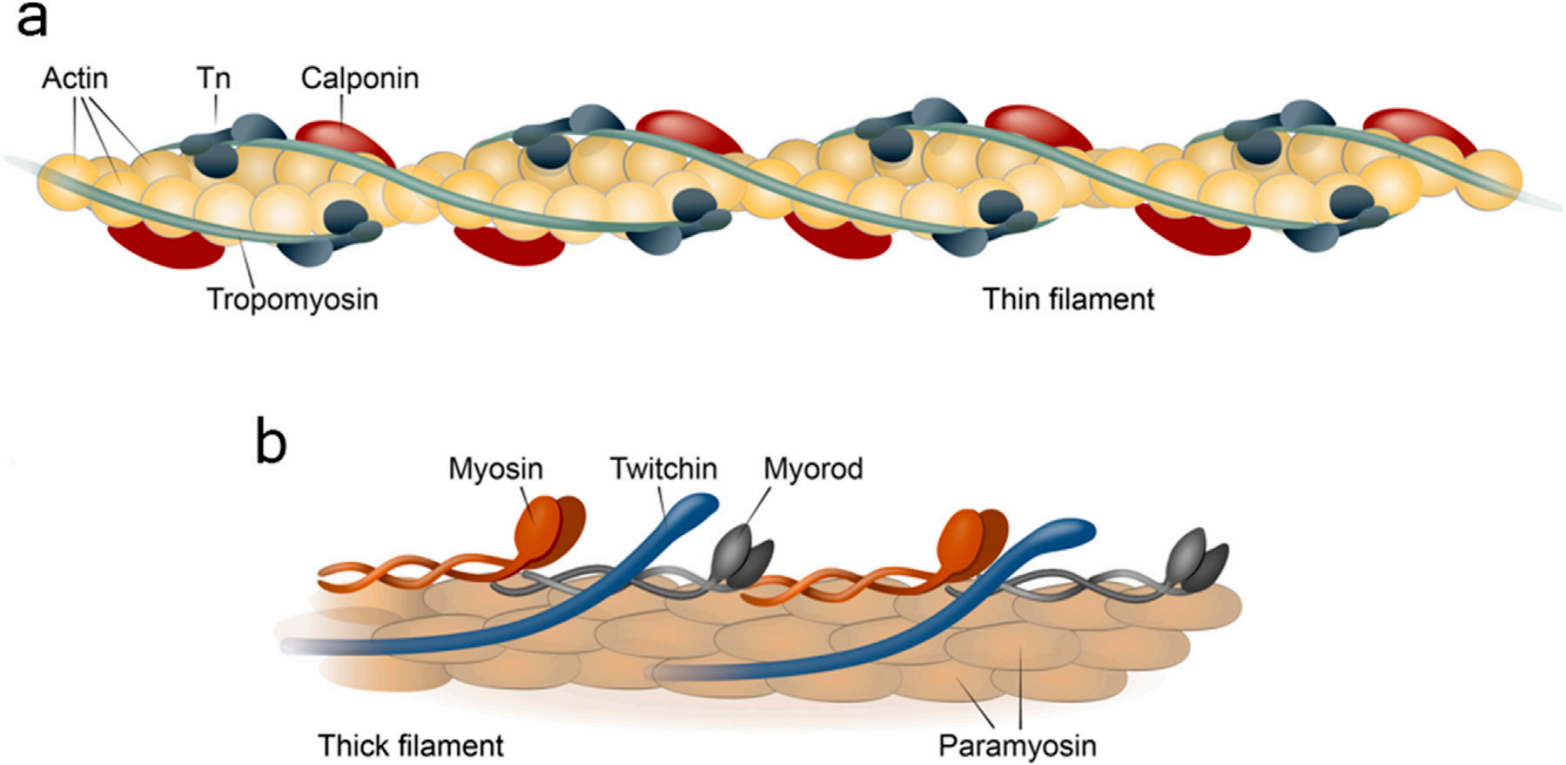
Structure of thin **(a)** and thick **(b)** filaments of the contractile apparatus of the catch muscle. Tn, tropoinin complex.

Like all other types of muscles, fibers of catch muscle include thick and thin filaments (Figure 2). Thick filaments of catch muscles are exceptionally large in size compared to those of other muscles, both in diameter (up to 75–100 nm) and in length (up to 50–100 µm) (Lowy and Hanson, 1962; Sobieszek, 1973). For comparison, thick filaments of striated muscles in the scallop *Mizuhopecten yessoensis* are approximately 20 nm in diameter and 1.8–2.0 µm in length (Elfvin et al., 1976). It is likely that such prominent sizes of thick filaments may be associated with the extremely high rates of force developed by the catch muscles. Compared to vertebrate skeletal muscles, where the emphasis is on the parallel contraction of many miniature sarcomeres, here, due to the size, the contact area between the counter-directional structures of the contracting subunits is enlarged. As a result, each subunit develops a significantly greater force. The increase in the size of thick filaments was possible due to their peculiar structure with the major part of filament occupied by a paracrystal of the protein paramyosin, not by the polymer myosin. The remaining proteins of thick filaments, including myosin, form a monolayer on its surface (Cohen et al., 1971; Hardwicke and Hanson, 1971; Szent-Györgyi et al., 1971).

##### 1.1.1.1 Paramyosin

Let us undertake a more detailed examination of the function and properties of the structural core of thick filament, the paramyosin protein. Firstly, it should be noted that paramyosin is not a unique catch muscle protein, as it has been found in thick filaments of all invertebrates studied (Elfvin et al., 1976; Hooper et al., 2008). However, in catch muscles, the level of paramyosin is multifold higher than in muscles that do not exhibit the catch properties. It can reach 60% of the total protein content (Levine et al., 1976a; Bennett and Elliott, 1981; Elliott and Bennett, 1982). The high paramyosin content of smooth muscles in bivalve molluscs is associated with their functional features (Ruegg, 1961; Ruegg, 1964; Lowy et al., 1964; Watabe and Hartshorne, 1990), in particular, with the strength of the tension they develop. Thus, the tensile stress produced by an intact adductor muscle is about 10–14 kgf/cm^2^ cross-section, while the tensile stress developed by vertebrate skeletal muscles is only about 3.5 kgf/cm^2^ (Twarog, 1967b). Such correlation is apparently due to the paramyosin core, which increases the length and thickness of thick filament and, therefore, the number of contacts of this filament with thin ones is also increased (Lowy et al., 1964). The only function of paramyosin is probably structural, because, despite numerous studies, and even the promotion of a separate hypothesis of catch state (Section 1.4) associated with this protein (Ruegg, 1961), no other its functions have been reported to date.

The paracrystal of paramyosin, also referred to as thick filament core, a structure resembling a two-dimensional net (Cohen et al., 1971; Szent-Györgyi et al., 1971; Castellani et al., 1983) formed through successive shifts of adjacent rows of protein molecules. These filaments have a 72.5 nm axial periodicity with a prominent 14.5 nm repeat (Cohen et al., 1971). The surface of the paramyosin polymer with surface proteins removed, stained for electron microscopic observations, shows a large-scale transverse ordering of the filaments: in the electron microscope a striking net array with a “checkerboard” appearance due to its grid/net-like structure could be seen in filaments from certain preparations (Szent-Györgyi et al., 1971). This pattern was termed the Bear–Selby net after the researchers who first described it (Bear and Selby, 1956). As for the molecular structure of paramyosin, paramyosin, is a fibrillar structural protein of thick filament 130 nm in length and 2 nm in diameter consisting of two polypeptide chains coiled into a superspiral (Kendrick-Jones et al., 1969). Both paramyosin chains are apparently identical and oriented in parallel (Weisel and Szent-Gyorgyi, 1975). The molecular weight of paramyosin varies depending on its source within a range of 200–226 kDa (Winkelman, 1976). Paramyosin is known for its exceptional resistance: one of the techniques to isolate it includes treating a muscle extract with ethanol (Szent-Györgyi et al., 1973). Nevertheless, it is important to note that the solubility of paramyosin depends on pH. As the pH value of the solution is decreased from 7.0 to 6.75, paramyosin loses its solubility and forms a paracrystalline structure. This transition has an inverse relationship with the ionic strength of solution (Johnson and Kahn, 1959). The influence of thick filament proteins on this transition undoubtedly needs to be studied. The strong dependence of paramyosin properties on these medium conditions should be taken into account when designing the medium for artificial muscle in which paramyosin is planned to be used. Probably, by regulating the expression of paramyosin in the tissue, it is possible to increase or decrease the size of individual thick filaments, changing the maximum force developed by the muscle.

In general, the fact that the mechanisms of formation of the structures of the contractile apparatus of the hindlimb muscle *in vivo* are still not studied deserves special discussion. While thin filaments are arranged in a similar way in all muscles due to the conservative actin structure, thick filaments of the locking muscles, as you have already realised, are much more complex than thick filaments of vertebrate skeletal muscles. Understanding the mechanism of assembly of such a structure is very important when creating synthetic muscles, and therefore, wherever possible, in this review we will focus on the polymers formed by proteins and how the properties of these polymers are determined.

As previously outlined, other thick filament proteins are situated on the surface of the paramyosin paracrystal, specifically: myosin (Szent-Györgyi et al., 1971), myorod (Shelud’ko et al., 1999), and twitchin (Shelud’ko et al., 2004; Funabara et al., 2007a). The structure, properties and function of these proteins are not fully understood at the moment; however, it is these proteins that appear to be involved in the realisation of the catch state and hence are of great interest for their application in biorobotics. Let us now examine each of these in greater detail.

##### 1.1.1.2 Myosin

The molecular weight of the catch muscle myosin is 450 kDa. Its structure generally resembles that of the myosin of other muscles, with its molecule consisting of two heavy chains (200 kDa each) and two light chains (15–30 kDa each). The heavy chains of myosin form two heads (12–20 nm each) and a tail 140 nm long (Flicker et al., 1981; Flicker et al., 1983). The tail is formed by α-helical segments of heavy chains woven into a double-spiral structure, and is responsible for binding to a thick filament (Harrison et al., 1971). Each of the myosin heads bears an actin-binding site and a site capable of hydrolysing ATP (Lowey et al., 1969). A small (8.5 nm) flexible α-helical rod, located between the tail and the myosin head, is responsible for conformational changes and bears sites for binding to light myosin chains (2 essential and 2 regulatory, 1 per head) (Houdusse and Cohen, 1996; Weeds and Lowey, 1971). Essential chains (17 kDa) are involved in maintaining the stability of the myosin molecule (Aguilar et al., 2010). Regulatory chains (20 kDa) contribute to the regulation of Mg^2+^-ATPase activity of myosin. These chains are involved in the formation of the Ca^2+^-binding domain, due to which the myosin from bivalve muscles is capable of self-regulation (Chantler and Szent, 1980). The purpose of this myosin ability is not entirely clear. Perhaps the calcium-sensitivity of myosin ATPase compensates for the lack of calcium regulation of thin filaments (section 1.1.2.3). Nevertheless, the use of this myosin in hybrid muscles may allow simplification of the structure of the contractile apparatus, since the regulation of thin filaments may be eliminated as unnecessary. It is noteworthy that myosin, which is devoid of regulatory chains, loses its capability of Ca^2+^-regulation and, simultaneously, the dissociated regulatory chains do not bear a Ca^2+^-binding domain (Asakawa et al., 1981; Bagshaw and Kendrick-Jones, 1979; Chantler and Szent-Gyorgyi, 1978; Jakes et al., 1976). The latter occurs in the presence of 10 mM EDTA at a temperature of 25°C–35°C (Chantler and Szent, 1980). Since the cleavage of the regulatory chain leads to a loss of control over myosin, the process of removing its regulatory chains has been termed desensibilisation. It should be noted that the EDTA treatment of myosin at a lower temperature leads to the cleavage of only one of its two regulatory chains (Szent-Györgyi et al., 1973), whereas the Ca^2+^-control of myosin is completely lost (Chantler and Szent, 1980; Jakes et al., 1976). Catch muscles contain two different isoforms of the myosin regulatory light chain, of which one is also present in bivalve striated muscles; the other isoform is specific to catch muscles and contains a phosphorylation site (Morita and Kondo, 1982). The role of this specific isoform is unknown. The presence of the calcium-sensitive domain is the major difference between the heavy chain isoforms of myosin of the catch muscle and myosin of vertebrate skeletal muscle. The other differences are generally no greater than those between the fast and slow skeletal muscle myosin isoforms of mammals (Weiss et al., 1999). At the same time, the ATPase rate of myosin of the catch muscles is much lower than that of skeletal muscle myosin, or myosin of the striated scallop muscle.

Myosin in bivalve smooth muscle is not the main component of the thick filament - its content in the contractile apparatus is 10 times lower than that of paramyosin (Vyatchin et al., 2019). Therefore, myosin in these muscles no longer has a structural function, but remains a functional unit of the thick filament. The very path of evolution to the creation of more massive thick filaments is a curious question. Probably, due to the peculiarities of the structure of polymeric myosin, the creation of filaments of comparable length and surface area using myosin alone is impossible. In addition, the very organisation of myosin on the surface of the paramyosin core still leaves many questions. The hypotheses in this regard are presented in sufficient detail in a recent review by Sobieszek (Sobieszek and Sobieszek, 2022), so we will not dwell on myosin polymerisation in detail.

A significant disadvantage of myosin of the catch muscles is the instability of molluscan myosin even during short storage periods (Barany and Barany, 1966; Kondo et al., 1979; Lehman and Szent-Györgyi, 1975). However, according to Chantler (Chantler, 1983), the instability of myosin is explained by the denaturation during preparation. Properly prepared bivalve myosin retains its activity and the ability to interact with actin in a Ca^2+^-dependent manner for at least 1 week. Moreover, myosin placed in 40% ammonium sulphate can be stored there for an indefinitely long time (Wallimann and Szent-Györgyi, 1981). The most convenient technique to prepare myosin for storage in ammonium sulphate was described by Chantler and Szent-Gyorgyi (Chantler and Szent-Gyorgyi, 1978). This technique is based on ammonium sulphate fractionation (Focant and Huriaux, 1976) which allows obtaining pure myosin within the shortest possible time. However, the relatively lower thermostability of this myosin and the low level of ATPase activity, in our opinion, reduce the value of this myosin in the creation of hybrid muscles.

##### 1.1.1.3 Myorod

**FIGURE 3:**
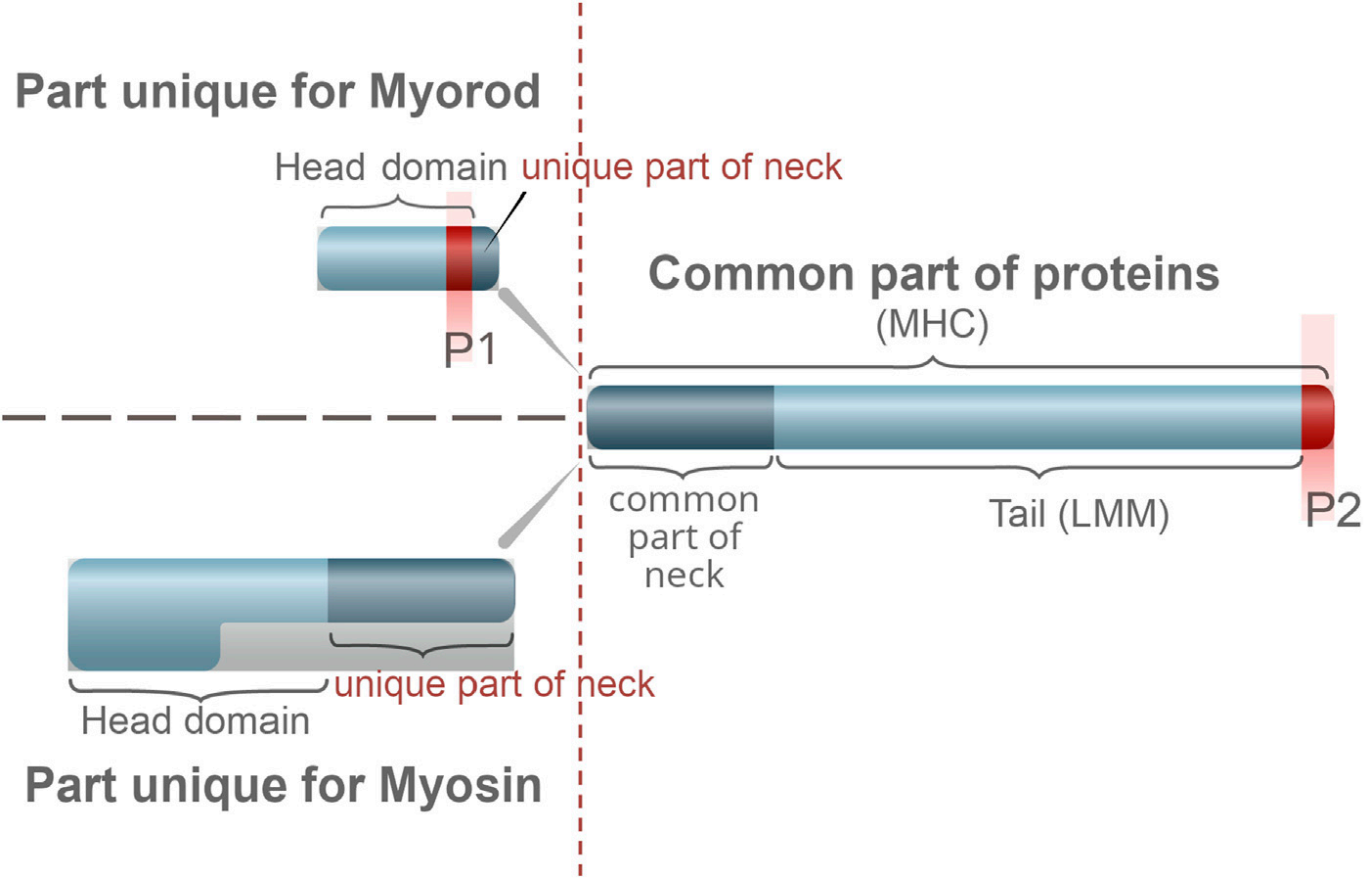
Comparison of the structure of catch muscle myorod and myosin. Similar part of the protein structure is shown on the right, unique ones on the left. The letter P indicates the sites of phosphorylation.

Myorod (also referred to as myosin rod protein, or catchin) is another surface protein of catch muscle thick filaments with a molecular weight of 220 kDa. This protein, first isolated from the adductor muscle of *Mytilus edilus* (Castellani and Cohen, 1988), turned out to be specific for bivalve catch muscles (Shelud’ko et al., 1999; Andersen et al., 2009; Matusovsky et al., 2017). Myorod was found in 20 out of 24 muscles of seven bivalve species studied, and its content in muscle is as high as that of myosin (Shelud’ko et al., 1999; Vyatchin et al., 2019; Shelud’ko et al., 1998). This protein is a product of alternative splicing of the myosin heavy-chain gene (Yamada et al., 2000); the substructure of myorod is similar (Figure 3) to the myosin substructure (Shelud’ko et al., 2002). However, in addition to the C-terminal “tail” part (830 residues) of myosin heavy chain, it has rather a unique N-terminal domain of 156 residues, not a pair of heads (Yamada et al., 2000; Yamada et al., 1997). The myorod consists largely of two polypeptides, 106 and 113 kDa (Shelud’ko et al., 1999; Shelud’ko et al., 2001). Domains having a high affinity for paramyosin are located in the C-terminal amino acid sequence of myorod, as in myosin, which allows it to interact with the core (McLachlan and Karm, 1983). Myorod is presumably integrated into the surface of the paramyosin core in the same way as myosin: its “tail” (C-rod) is located on the surface of thick filaments, while the “head,” like the myosin heads, protrudes into the interfilament space (N-terminal end) (Szent-Györgyi et al., 1971; Shelud’ko et al., 1999; Shelud’ko et al., 2002; Vibert et al., 1993). *In vitro*, myorod forms polymers that substantially differ from myosin polymers despite the identity of the sites determining the polymerization degree of these proteins (Shelud’ko et al., 2001; Matusovskaya et al., 2004; Matusovsky et al., 2005). This indicates the effect of the myorod head on the functional state of its tail (Matusovsky et al., 2005).

Differences in the properties of polymeric myosin and myorod should be taken into account when using their solutions and suspensions, or developing hybrid muscles, for example, devoid of paramyosin. In fact, the native myorod has an unusual property: a pronounced thixotropy, which is associated with its high structural viscosity (Shelud’ko et al., 2001). The viscosity of myorod is by an order of magnitude higher than that of the polymer myosin. With a variation of ionic strength, the spatial structure changes: lateral aggregates are formed at 50 mM KCl; long filamentous polymers are formed at 100 mM KCl; and short polymers are observed at 150 mM KCl. It is likely that molecules of this protein can interact laterally with each other (Matusovsky et al., 2005).

Similar filaments are formed by a proteolytic fragment of myorod after detachment of the unique sequence. In addition, the unique sequence detachment leads to a sharp variation in the rheological properties of myorod (viscosity and thixotropy). Without the unique sequence, the viscosity of hydrogen is no higher than that of HMM or LMM, and thixotropy is absent. On the other hand, modification of the amino acid Cys722 by N-ethylmaleimide in the tail part of myorod completely inhibits its polymerization, but imparts it the ability to aggregate in the presence of Mg^2+^ (Matusovskaya et al., 2004). This property has been reported only for the native myorod. All the above facts indicate a strong effect of a small unique domain on the properties of the rod domain and, accordingly, a potentially high regulatory capacity of the N-terminal unique domain of myorod. This assumption was confirmed after the discovery of phosphorylation of the N-terminal unique domain of myorod (Sobieszek et al., 2006). In solution, myorod can form copolymerized filaments with myosin (Yamada et al., 2000; Shelud’ko et al., 2001; Sohn et al., 1997). In such a polymer, myorod can be phosphorylated by kinases associated with myosin of bivalve smooth muscle (Matusovsky et al., 2010a). Moreover, myorod can be phosphorylated by myosin light-chain kinase (and probably by twitchin of bivalve smooth muscles) from the vertebrate smooth muscles at Thr-141 position (Sobieszek et al., 2006). This indicates the similarity of its N-terminal head domain with the regulatory light-chains of myosin. The phosphorylation region (Thr-141) is localized in the N-terminal unique domain of myorod, which may indicate the regulatory role of phosphorylation in protein functioning. Thr-141 is assumed to be involved in actin-myorod interaction, although it was previously suggested that myorod does not interact with actin *in vivo*, since its N-unique part is shorter than the myosin part (Yamada et al., 2000; Shelud’ko et al., 2001). Experimental data on the precipitation of a mixture of actin with artificially synthesized peptides of an N-terminal unique segment of myorod have shown the probability of such an interaction. The addition of non-phosphorylated N-terminal peptides led to aggregation and precipitation of actin filaments during low-speed centrifugation. Peptides in which phosphorylation was mimicked (Asp at position Thr-141) had no such effect (Matusovsky et al., 2011). However, as fluorescence microscopy showed, actin forms dense bundles after the addition of hydrogen peptides (Matusovsky et al., 2011). This suggested that phosphorylation is essential for the functional role of myorod and can also influence the actin–myosin interaction. Phosphorylation of the N-terminal domain of myorod increases the actin-activated Mg^2+^-ATPase activity of myosin (Matusovsky et al., 2015).

In general, a conclusion can be made that myorod can interact with most proteins of the catch muscle contractile apparatus: paramyosin, myosin, twitchin, and polymer actin (Shelud’ko et al., 1999; Shelud’ko et al., 2001; Matusovsky et al., 2010a; Matusovsky et al., 2011). However, the functional consequences of these interactions are not entirely clear. Given the significant content of myorod in the catch muscle and its ability to tightly bind actin (Vyatchin and Dyachuk, 2024), it is likely that this protein is responsible for the formation of catch-linkages. Consequently, it is myorod that can be used in the creation of hybrid muscles, to realise the ability for catch state function. This is also favored by the outstanding physicochemical properties of this protein. Although myorod is made up of more than 75% the rod part of myosin, its solubility and resistance to high temperatures differ: myorod is a thermostable protein, and one of the approaches to its purification is boiling (Shelud’ko et al., 1999). This resistance to temperature may prove useful in biorobotics, to create mechanisms capable of operating under unfavourable environmental conditions.

##### 1.1.1.4 Twitchin

**FIGURE 4:**
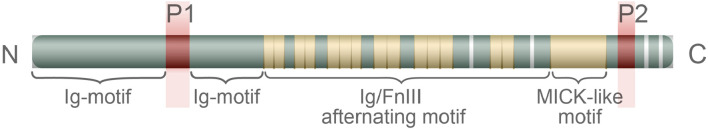
The structure of the twitchin of the catch muscle. The letters P1 and P2 indicate the sites of phosphorylation. The letters N and C to the left and right of the diagram indicate the corresponding ends of the proteins.

Twitchin is the last of the thick filament proteins under consideration. It is a giant protein (530 kDa) attributed to the protein kinase superfamily. It belongs to the “CAMK (Ca^2+^/calmodulin-dependent protein kinase) Ser/Thr protein kinase” family. The latter comprises giant proteins consisting of repeated copies of immunoglobulin (Ig) and fibronectin-like (Fn III) domains (Figure 4), including titin (Benian et al., 1996). Proteins of the titin family have long, stretched molecules and contain a serine/threonine kinase domain near the C-end, which is homologous to the catalytic region of the myosin light-chain kinase (MLCK). With so many binding centres, it’s not surprising that twitchin is apparently a multi-functional protein that can interact with many proteins such as bivalve myosin (Yamada et al., 2001; Shelud’ko et al., 2007), paramyosin, myorod, thin filaments, actin (Shelud’ko et al., 2007), and, probably, calponin (Matusovsky et al., 2017). However, the exact function of this protein in the contractile apparatus has not been determined, although it is clear that with such a large number of molecular interactions, twitchin probably performs far more than one task in the cell. Furthermore, this protein is clearly one of the functional players involved in the formation of catch state, as described below. Such a protein could probably have many different applications in synthetic muscles. For this reason, let us dwell on its structure in more detail.

The complete amino acid sequence of the twitchin of the mussel *Mytilus galloprovincialis* has been determined (Funabara et al., 2003). The sequence of this protein includes 24 immunoglobulin repeats, 15 fibronectin type III repeats, a small PEVK-like domain, and a kinase domain having high homology with MLCK (Funabara et al., 2003). The belonging of twitchin to the protein kinase superfamily and the detection of the MLCK domain in its sequence suggest its ability to act as a kinase that can be targeted at both myosin and myorod. In turn, twitchin can be phosphorylated and dephosphorylated at two sites detected through proteolytic cleavage of the phosphorylated twitchin molecule (Funabara et al., 2003). These sites are referred to as D1 (Ser-1075) and D2 (Ser-4316), and are located at opposite ends of the molecule. Site D2 is located behind the kinase domain between 21 and 22 Ig domains; site D1 is localized in the N-terminal part between 7 and 8 Ig domains. Phosphorylation of twitchin can be carried out *in vitro* using cAMP-dependent protein kinase A (Siegman et al., 1997; Siegman et al., 1998; Yamada et al., 2004a; Béjar and Villamarín, 2006); dephosphorylation, using Ca^2+^/calmodulin-dependent Ser/Thr phosphatase 2В (PP2B) (Siegman et al., 1997; Siegman et al., 1998; Yamada et al., 2004a; Klee et al., 1988; Castellani and Cohen, 1992). It should be noted that phosphorylation of twitchin site D2 is possible only after complete phosphorylation of D1 (Funabara et al., 2003). Nevertheless, the finding of a PEVK-like domain in the twitchin structure is of particular interest. A similar site has also been found in the titin sequence, where it provides the elasticity of the sarcomere, acting like a molecular spring, i.e., unwinding and stretching under high loads, and winding and shortening at rest (Linke, 2000; Kulke et al., 2001; Granzier and Labeit, 2002). It would be a mistake to assume that the presence of the PEVK domain in twitchin indicates an overlap between the functions of twitchin and titin. For example, twitchin is: several times shorter than titin; localises directly to the surface of thick filaments; does not form separate filaments; and is unable to polymerise (hence the omission of this section in this chapter).

Nevertheless, there is every reason to believe that under tension this domain activates the kinase activity of the twitchin MLCK domain. The MLCK domains of titin have a similar ability (Gräter et al., 2005).

Finally, the role of twitchin in regulating the catch state need to be discussed. The phosphorylation of twitchin using cAMP-dependent protein kinase A has been shown to relax the muscle from the catch state (Siegman et al., 1997; Siegman et al., 1998; Yamada et al., 2004a; Béjar and Villamarín, 2006) and, as was shown later, lead to dissociation of twitchin–actin complexes (Shelud’ko et al., 2004; Méndez-López et al., 2012). Thus, the twitchin phosphorylation does not affect its interaction with the thick filament proteins, but significantly decreases its interaction with F-actin and thin filaments (Shelud’ko et al., 2004; Shelud’ko et al., 2007; Funabara et al., 2007b; Butler et al., 2010). The dephosphorylation of twitchin by PP2B restores the muscle’s ability to maintain high tension (Siegman et al., 1997; Siegman et al., 1998; Yamada et al., 2004a; Klee et al., 1988; Castellani and Cohen, 1992). Therefore, during the catch state, when the muscle maintains a high tension, twitchin is in a dephosphorylated state. The phosphorylation of twitchin and the associated process of muscle relaxation from the catch state occurs within 30 s after the addition of cyclic AMP to skinned muscle fibers or serotonin to native muscle (Siegman et al., 1997). Clarification of the effect of twitchin phosphorylation and dephosphorylation on the contractile apparatus has led to the hypothesis of “twitchin linkages” (Shelud’ko et al., 2007). At the moment, it is clear that twitchin is much inferior to the other proteins of thick filaments in its content in muscle (ratio is 100 paramyosin: 10 myosin: 10 myorod: 1 twitchin). At this content, the protein is certainly not able to fulfil the role of the catch cross-linkage alone (Vyatchin et al., 2019). Nevertheless, experiments with the introduction of sufficient amounts of this protein into the contractile apparatus of vertebrate skeletal muscles have shown good results (Avrova et al., 2009) - therefore, this protein may be sufficient to give synthetic muscles the ability to catch. The effect of twitchin on the functioning of the calcium-regulatory system of thin filaments deserves separate attention (Avrova et al., 2010), but we will address this issue further in the corresponding Section 1.1.2.3.

#### 1.1.2 Structure of thin filaments of catch muscle

In order to understand the structure of the contractile apparatus of the catch muscles (for the purpose of its further reproduction), it is worthwhile to briefly go over the proteins of thin filaments and their organisation. The bivalve catch muscle is smooth and differs little from other smooth muscles in its structure. As in other smooth muscles, thin filaments of the contractile apparatus are arranged into an irregular pattern, attached to dense bodies (Figure 1), and their number is much greater than the number of thick filaments (Sobieszek, 1973). The thin to thick filaments ratio is about 15: 1, whereas in striated muscles it is 2: 1 (Murphy, 1976).

The protein structure of thin filaments is quite typical, with the major protein being actin (Figures 1, 2). Tropomyosin, troponin complex (Vyatchin et al., 2015), and calponin (Dobrzhanskaya et al., 2013) are located on the surface of actin. However, the protein caldesmon, characteristic of vertebrate skeletal muscles and involved in Ca^2+^-dependent regulation together with calponin, is absent (Dobrzhanskaya et al., 2013) despite some evidence (Bennett and Marston, 1990). Let’s take a closer look at the characteristics of these proteins and their potential benefits for synthetic muscle.

##### 1.1.2.1 Actin

Actin is a protein expressed in almost all organs and tissues of animals and plants (Pollard et al., 1986; Hooper and Thuma, 2005). The amino acid sequence of actin has a high degree of conservativeness, being one of the highest among eukaryotes (Hooper and Thuma, 2005; Elzinga et al., 1973; Vandekerckhove and Weber, 1978; Vandekerckhove and Weber, 1979). The differences in the amino acid sequence between different actins, both within the same species and interspecific, are extremely insignificant and amount to no more than 25 amino acid substitutions. The catch muscle actin is no exception (Patwary et al., 1996). Therefore, the question of actin interchangeability begs the question, and in biochemical work, the difference between actins is often neglected. Despite this, a southern blot analysis has shown the presence of up to 15 actin genes in the genome of *Placopecten magellanicus*. Such a diversity of isoforms must have significant reasons, therefore, the use of one single actin isoform in synthetic muscle is an interesting idea that should, however, be implemented with caution. The study of the properties of different actin isoforms has been complicated by the fact that until recently, there has been no method for obtaining globular actin of smooth catch muscle, which is capable of repolymerization (Shelud’ko et al., 2016). It should be noted that early electrophoresis-based studies did not reveal differences in actin motility either for a wide range of bivalve species (Margulis and Pinaev, 1976) or between different tissues within the same species studied (Margulis et al., 1982). A detailed study of the properties of catch muscle actin became possible after the discovery of the method for non-denaturing isolation of molluscan actin (Shelud’ko et al., 2016; Girich et al., 2017). This allowed the polymerisation dynamics, viscosity and activation capacity of catch muscle actin to be compared with those of vertebrate skeletal muscle actin. It was found that the activating abilities and polymerization dynamics of purified globular actins of the catch muscle and vertebrate skeletal muscles are almost identical. However, the characteristic viscosities of the actins measured by the falling ball method differ 4–8-fold. The authors attribute these differences to the degree of actin purification from capping proteins. While actin is purified of a supposed impurity, the differences in actin viscosity decrease (Girich et al., 2017). Understanding the differences and functions of the multiple actin isoforms present in the catch muscles requires further careful comparison of the properties of these proteins.

##### 1.1.2.2 Tropomyosin

Tropomyosin is a fibrillar protein located on the surface of thin filaments and involved in the formation of the calcium-regulatory system of thin filaments. Like actin, this protein demonstrates sufficient conservativity of structure and properties. The study of the paracrystal of tropomyosin showed that during polymerization its successive molecules overlap head-to-tail (Murakami et al., 2008). The overlap length is 9 amino acid residues (Lehman et al., 2015; Phillips et al., 1979). Tropomyosin paracrystals from different sources are very similar, e.g., between the oyster *Crassostrea commercials* and the abalone *Notohaliotis ruber* (Millward and Woods, 1970; Woods and Pont, 1971). Significant similarities are also observed between tropomyosin paracrystals from different tissues of the same animal, e.g., from the adductor muscle and the foot of the mussel *Anodon pacifica* (Tsao et al., 1965). Tropomyosin paracrystals from all these muscles show a 40-nm striation periodicity, which can be used to estimate the length of the tropomyosin molecule. The same striation pattern is characteristic of paracrystals of the skeletal muscle tropomyosin.

The catch muscle tropomyosin resembles the vertebrate tropomyosin in a variety of physicochemical properties. Thus, the molecular weight of tropomyosin ranges within 28–34 kDa (Woods and Pont, 1971). Tropomyosin is soluble in high ionic strength solution, polymerizing with a decrease in ionic strength to a level below 0.1 М (Sousa and Farah, 2002; Tsao et al., 1951). Polymerisation can be detected by a significant increase in the viscosity of the solution. The ability of tropomyosin to polymerize in low ionic strength solution is due to its C-terminal sequence. Thus, if the terminal 9-amino acid sequence is cleaved by carboxypeptidase A, the resulting tropomyosin will be unable to polymerise and interact with actin, even if there is an excess of magnesium in the solution (Cho et al., 1990; Mak and Smillie, 1981).

The tropomyosin denaturation profile, observed as the molar ellipticity of the solution at 222 nm (characterizing the content of α-helices in the solution), showed that the thermal stability of tropomyosin from muscles of some bivalves is close to that of vertebrate tropomyosins (Woods, 1976). At the same time, the vertebrate and bivalve tropomyosins have significantly different temperatures of dissociation from actin. Thus, the tropomyosin of the scallops *Argopecten irradians* and *Placopecten magellanicus* dissociates from actin at a temperature of 25°C and above or at a high ionic strength (0.6 M NaCl and 2 mM Mg-ATP), while the dissociation of skeletal muscle tropomyosin occurs at 47°C (Nevzorov et al., 2011). This is probably explained by the difference in the overlapping regions of neighbouring tropomyosins in thin filaments of scallops and vertebrates (Chantler and Szent, 1980; Newman and Carlson, 1980). It is important to note here that the general thermal stability of the tropomyosin from the scallop *Argopecten irradians* is somewhat lower than that of the tropomyosins of other bivalves (Woods, 1976). These data indicate that scallop tropomyosins are less suitable for use in synthetic muscle than proteins from other muscles.

For the development of hybrid synthetic muscles, the representative history of research on the inhibitory activity of molluscan smooth muscle tropomyosin should be helpful. One of the most noteworthy properties of catch muscle tropomyosin is its ability to inhibit the ATPase activity of myosin in the absence of regulatory proteins (Nishimura et al., 1997; Nishita et al., 1997). The authors who discovered this effect of tropomyosin assumed this unusual ability of tropomyosin to be associated with the specific features of the Ca^2+^-regulatory system of catch muscle thin filament. These features indicated that this system belongs to the “activator type,” in contrast to the regulatory system of thin filaments of vertebrate skeletal muscles belonging to the “inhibitory type.” However, this turned out to be caused rather by the combination of proteins from various sources within the framework of one experiment that by the type of regulatory system of thin filaments. Thus, as was found later, the catch muscle tropomyosin inhibits specifically the ATPase activity of skeletal muscle myosin, but not the catch muscle myosin (Shelud’ko et al., 2015). Nevertheless, as the authors of the concept of the regulatory system of inhibitory type thin filaments previously assumed, the addition of the catch muscle troponin complex to tropomyosin normalized its properties (Vyatchin et al., 2015). Another similar discovery was the lack of the effect of the catch muscle tropomyosin on the viscosity of skeletal muscle actin. However, the viscosity of the catch muscle actin increased in the presence of tropomyosin (Girich et al., 2017).

As for the functional role of tropomyosin in the catch muscle, it is assumed to perform the same functions as in others: structural (increases the rigidity of actin filaments) and regulatory (being part of the calcium-regulatory system of thin filaments) (Nevzorov et al., 2011). Nevertheless, its role in the catch state mechanism cannot be ruled out as well. Thus, twitchin has been shown to be able to “freeze” the catch muscle tropomyosin in a blocked position that prevents strong myosin to actin binding (Avrova et al., 2010). The authors indicate that such a freeze may be necessary to maintain a low level of myosin ATPase during the state of catch state.

##### 1.1.2.3 Troponin complex

Troponin, first obtained by Ebachi (Ebachi et al., 1968), is a key protein in the actin-associated regulation of striated muscles, both vertebrates and invertebrates. It binds calcium released from the sarcoplasmic reticulum under the effect of a nerve impulse, thus, activating contraction (Green, 1987). The troponin regulation is characteristic of striated muscles of both vertebrates and invertebrates (Lehman, 1976). Due to the relative familiarity of the mechanism underlying troponin function, this type of regulation may well be utilised in the creation of artificial muscles. Moreover, it was recently confirmed that troponin regulation of thin filaments is characteristic not only of striated muscles of molluscs, but also of their smooth muscles (Vyatchin et al., 2014). The structure of the discovered thin filament calcium-regulatory system of molluscan muscles is little distinguishable from that of vertebrate skeletal muscles. For example, the ratio between actin, tropomyosin, and troponin complex in scallop muscles is reported to be the same as that known for skeletal muscles, 7: 1: 1 (Lehman et al., 1980). The ratio of troponin components in the troponin complex is equimolar (Nishita et al., 1997; Ojima and Nishita, 1986). The troponin components of both types of scallop muscles correspond to the troponin components in vertebrates in their both separate and combined effect on the contractile model (Nishita et al., 1997; Ojima and Nishita, 1986; Ojima and Nishita, 1992). Moreover, the troponin components of these muscles turned out to be interchangeable (Goldberg and Lehman, 1978; Ojima and Nishita, 1988). However, there are some differences between the vertebrate and bivalve troponins. Thus, the C-component of scallop troponin, unlike the vertebrate troponin C, has one Ca^2+^-binding domain, not two. This is true for troponin C from both smooth and striated scallop muscles (Nishita et al., 1997; Ojima and Nishita, 1986). Despite all the above facts, some doubts about the involvement of troponin in the calcium regulation in scallop smooth muscles still persist (Chantler, 2011).

As regards the occurrence of the troponin regulation beyond the Pectinida group, it should be noted that the presence of troponins in catch muscles has also been reported for other taxonomic groups of bivalves (Ostreida and Venerida). It is worth emphasizing, however, that their presence is only nominal, as evidenced by either electrophoretic (Lehman, 1981) or genetic analyses (Funabara et al., 2013; Umasuthan et al., 2013). The exception is the Mytilida order, where fully functional troponins were found, as those in scallop smooth muscles (Vyatchin et al., 2015). Nevertheless, the troponin content of the Mytilida catch muscle (1 troponin: 2 tropomyosins) is twice as low as that of Pectinida striated muscles (1 troponin: 1 tropomyosin). The authors explain this by the presence of smooth muscle filaments from both contractile and cytoskeletal compartments of the cell in the analysed preparation. Anyway, with such a content, troponins obviously cannot provide full regulation of the entire pool of catch muscle thin filaments. Probably, only partial regulation of thin filaments by the troponin complex is the reason for the presence of regulation of thick filaments (calcium-sensitive myosin) in these muscles.

##### 1.1.2.4 Calponin

The last protein of the thin filaments of the catch muscle to be considered is the least studied calponin-like protein. Additional studies are required to understand its function and further application in biorobotics. Calponin belongs to the family of actin-binding proteins (Masuda et al., 1996; Applegate et al., 1994). Two its isoforms, 34 and 40 kDa, were found in the catch muscle of the mussel *C. grayanus*. To date, only one calponin isoform, weighing 40 kDa, has been studied in detail (Matusovsky et al., 2017; Dobrzhanskaya et al., 2010; Sirenko et al., 2012; Sirenko et al., 2013). It has been shown that calponin is a water-soluble, thermostable, basic protein capable of interacting with actin by inhibiting the Mg^2+^-ATPase activity of actomyosin regardless of the concentration of free calcium. Apparently, the inhibition mechanism is based on the ability of calponin to suppress the formation of strong forms of binding between the myosin subfragment-1 and actin, transferring them to a weaker form of binding (Sirenko et al., 2016). These results are consistent with the studies that have shown the calponin of vertebrate smooth muscles to have an effect on the conformation of actin, thereby regulating the myosin to actin binding (El Mezgueldi and Marston, 1996).

Calponin can be phosphorylated by endogenous kinases, except protein kinase A that can phosphorylate twitchin (Dobrzhanskaya et al., 2010). Like the vertebrate calponin having two sites for binding actin and is, therefore, capable of aggregating it (Kolakowski et al., 1995; Lu and Chalovich, 1995; Tang and Janmey, 1996), the catch muscle calponin is also capable of this (Dobrzhanskaya et al., 2013). The amino acid sequences of the catch muscle calponin-like protein are known for three bivalve species (Matusovsky et al., 2017; Méndez-López et al., 2012; Funabara et al., 2015). The expression of the calponin gene has been studied: it is most expressed in the mantle, anterior, and posterior adductor muscle (Matusovsky et al., 2017; Umasuthan et al., 2013; Wang et al., 2016). As an analysis of the primary sequence has shown (Matusovsky et al., 2017), the protein molecule contains potential binding sites of protein kinase C, protein kinase A, and Ca^2+^/calmodulin-dependent protein kinase II. Three acetylated residues have also been found, of which one (Asp127) is located in highly conserved CH-region of calponin-like protein (CaP-40). The authors suggest that a modification of this residue (Asp127) can seriously affect the functioning of the protein and possibly even regulate its compartment localization (Parker et al., 1994). Another result of the primary sequence analysis was the finding of a possible site of interaction between calponin-like protein and twitchin, one of the important components of catch muscle thick filaments (Matusovsky et al., 2017).

As regards the functional role of the catch muscle calponin, it should be noted that the function of even more deeply investigated vertebrate calponin still remains unclear. Calponins are suggested to be involved in various biological processes such as the regulation of contraction in smooth muscles (Winder and Walsh, 1996), intracellular signalling (Menice et al., 1997), and the organization of the actin cytoskeleton in smooth muscle and non-muscle cells (North et al., 1994). Therefore, the potential for the use of calponin in artificial muscles is certainly there, but not yet obvious.

### 1.2 Ratio between the proteins of the catch muscle contractile apparatus

An important aspect for the proper functioning of the contractile apparatus is its spatial organisation, often related to the number of certain structures and proteins. Knowledge of the relationships between them can help in the design of artificial muscles. For the catch muscle we are considering, these ratios are quite unique: ratio between thin and thick filaments of the catch muscle contractile apparatus (Sobieszek, 1973) (15: 1) is strikingly different from the ratio existing in striated muscles (2: 1) (Murphy, 1976). As for the relationship existing between specific proteins of the contractile apparatus, the general estimation of the ratio existing between the proteins of smooth catch muscle filaments was made only for Mytilida (Vyatchin et al., 2019; Vyatchin et al., 2015). The ratios were determined by densitometry of electrophoretic gels with preliminary calculation of the coefficient of dye binding to each of the proteins under study. The resulting ratio for thin filament (adjusted for the molecular weight of the protein and with the dye-binding coefficient taken into account) was as follows: 14 Аctin: 2 Тropomyosin: 1 Тroponin. The ratio for thick filament proteins, calculated with all necessary adjustments taken into account, was as follows: 100 Paramyosin: 10 Myosin: 10 Myorod: 1 Twitchin.

### 1.3 Actualization of the catch state hypotheses

**FIGURE 5:**
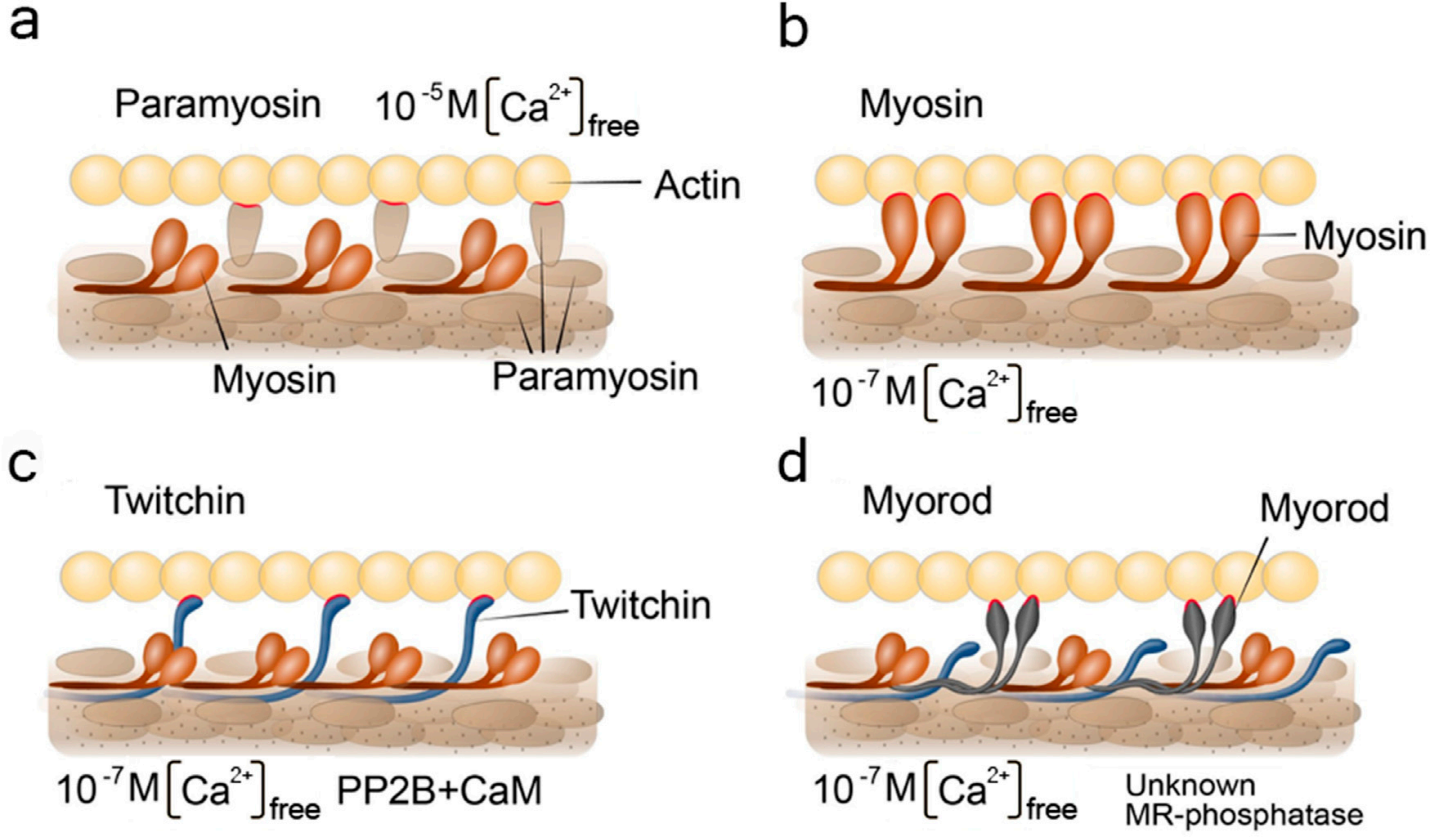
Schemes visualizing pre-existing hypothesis of the catch state. Under the letter **(a)** paramyosin hypothesis; **(b)** myosin hypothesis; **(c)** twitchin hypothesis; **(d)** myorod hypothesis. The figures indicate the conditions for the content of free intracellular calcium and the need for the presence of enzymes. CaM, calmodulin; PP2B, PP2B phosphatase; unknown MR-phosphatase, indicate an unknown phosphatase that phosphorylates myorod under unidentified conditions. Contacts of thick filament proteins with actin are highlighted in red.

Although only one hypothesis explaining the mechanism of the catch condition is still valid (Vyatchin and Dyachuk, 2024), in this section we would like to briefly mention other hypotheses that have already lost their relevance and summarise the actual data. Some of these hypotheses (of paramyosin-, myosin- and twitchin- links) are illustrated in Figures 5a-d. This may inspire synthetic muscle builders with ideas on how some useful muscle states can be realised in practice.

A similar attempt was recently made in a review by H. Sugi with co-authors, published a few years earlier (Sugi et al., 2020). However, it should be noted that the catch state formation pattern including the formation of a trimeric complex consisting of twitchin, myosin, and actin in the state of catch state, as reported in the Sugi’s review, seems to be erroneous. The ideas mentioned by the authors became a basis for a modernized version of the “linkage” hypothesis (Lowy et al., 1964; Lowy, 1953). According to it, the formation of a myosin cross-linkage between thin and thick filaments was assumed to depend on the state of twitchin phosphorylation (Butler et al., 2001; Funabara et al., 2001; Tsutsui et al., 2005; Yamada et al., 2004b). In particular, the dephosphorylated twitchin would give the catch properties to myosin cross-linkages, while the phosphorylated would not change the cycle of cross-linkages (Funabara et al., 2003; Siegman et al., 1997; Siegman et al., 1998; Butler et al., 2001; Funabara et al., 2001). This hypothesis (Figure 5b) turned out to be untenable in 2010, when S. Galler with co-authors proved that myosin does not interact with thin filaments in the state of catch state. This was shown by blocking myosin heads with inhibitors (Galler et al., 2010).

Another, recently relevant, hypothesis of the catch state was the “twitchin–actin linkage hypothesis” (Figure 5c). This hypothesis postulated that the twitchin protein of thick filament plays both a regulatory and a loadbearing role responsible for both regulation and maintenance of the catch state (Shelud’ko et al., 2004; Shelud’ko et al., 2007). It became possible to evaluate the validity of this hypothesis after measuring the exact twitchin content of catch muscle, followed by a reconstruction of the contractile apparatus model that took into account the estimated protein ratios (Vyatchin et al., 2019). In such a contractile model, the authors found that twitchin cannot prevent the onset of “relaxation” at its “natural” content (1 twitchin: 10 myosins). Nevertheless, upon clarification of the “natural” ratio between the proteins of the catch muscle contractile apparatus, it was found that the amount of myorod in this muscle is equimolar to the myosin amount. The reliably confirmed data on the substantial content of myorod and the refutation of the “twitchin linkages hypothesis” makes the hypothesis of “myorod bridges” more convincing (Matusovsky et al., 2011).

**FIGURE 6:**
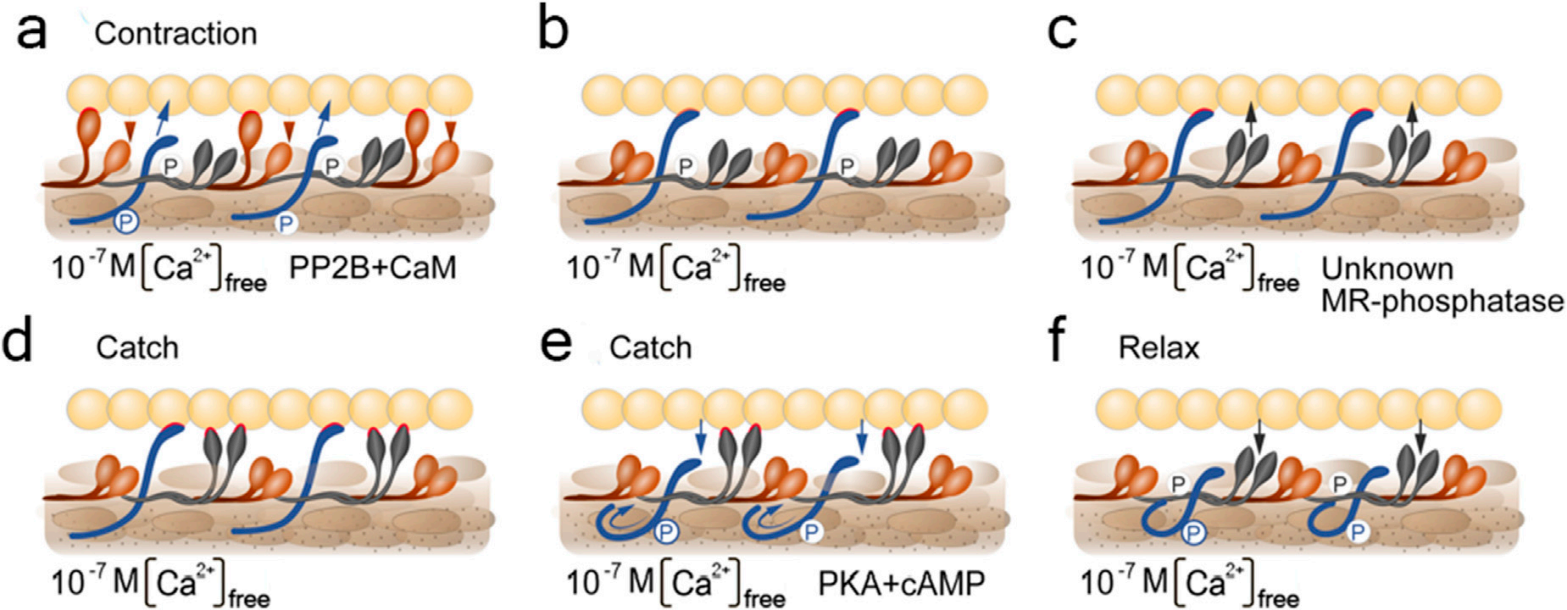
Scheme visualizing the cooperative hypothesis of the catch state. Different stages marked with letters **(a–f)**. Stage under letter **(a)** corresponds contraction; **(d)** and **(e)**–catch state; **(f)**–relaxation. Under the figures indicated the conditions for the content of free intracellular calcium and the need for the presence of enzymes. CaM, calmodulin; PP2B, PP2B phosphatase; PKA, protein kinase A; unknown MR-phosphatase, indicate an unknown phosphatase that phosphorylates myorod under unidentified conditions. Contacts of thick filament proteins with actin are highlighted in red. The arrows indicate emerging and disappearing protein-protein interactions. The circled letters P shows the phosphate residue transferred to the protein.

Historically, according to this hypothesis, as in all previously mentioned, it was assumed that a single protein plays a key role in the catch state – and this time it was myorod (Figure 5d). However, with modern data presented in this review, we can update this hypothesis and expand it to the cooperative one (Figure 6). In a state of relaxation (low calcium concentration), myorod and twitchin, both being phosphorylated, do not interact with actin (Figure 6f). In this case, thin filaments are in a state blocking the interaction with myosin, which is caused by own calcium-regulatory system (not shown in the figure) (Shelud’ko et al., 2007). Upon initiation of contraction, the cholinergic excitation induces a short-term increase in intracellular calcium levels (Figure 6a), thus, activating the calcium-regulatory systems of thin and thick filaments (myosin initiates active contraction). In parallel, calcium binding to calmodulin activates PP2B phosphatase, which dephosphorylates twitchin (Yamada et al., 2004a), leading to the formation of twitchin crosslinks (Figure 6b). The process of this links formation probably starts while intracellular calcium concentration been increased (Figure 6a) and finished only after decrease in calcium level (Figure 6b). At the same time, an unknown myorod phosphatase (Matusovsky et al., 2011) is also activated (Figure 6c), which leads to the formation of myorod crosslinks (Figure 6d). It should be noted that myorod links formation takes place precisely after or simultaneously with a decrease in calcium concentration, otherwise it would prevent the stage of active contraction. As a result, three processes are run in parallel: the active muscle contraction; the formation of twitchin (regulatory) linkages with actin and dephosphorylation of myorod. The latter leads to the decrease in the actin-activated Mg^2+^-ATPase activity of myosin (Matusovsky et al., 2010b), and the formation of myorod–actin links. It is noteworthy that the formation of twitchin links apparently leads to the end of the active contraction stage, possibly even despite the high concentration of intracellular calcium. This happens due to twitchin linkages “freezes” tropomyosin in the position blocking the access of myosin to actin (Avrova et al., 2010), thereby ending the active stage of contraction (and the expenditure of ATP by myosin). The formed myorod and twitchin linkages hold thin and thick filaments relative to each other, which creates the state of catch state. This lasts until cAMP is released, activating protein kinase A, which phosphorylates twitchin (Shelud’ko et al., 2004). Phosphorylation of twitchin detaches the links it has formed with actin, simultaneously activating the MLCK domain of twitchin (Figure 4), which, in turn, is apparently capable (Funabara et al., 2003) of myorod phosphorylation (Figure 6e). Phosphorylation of twitchin and myorod releases the calcium-regulatory system of thin filaments from the block state, and detachment the links formed by these proteins with actin, which in turn leads to the onset of relaxation (Figure 6f).

## 2 Development of catch muscle

**FIGURE 7:**
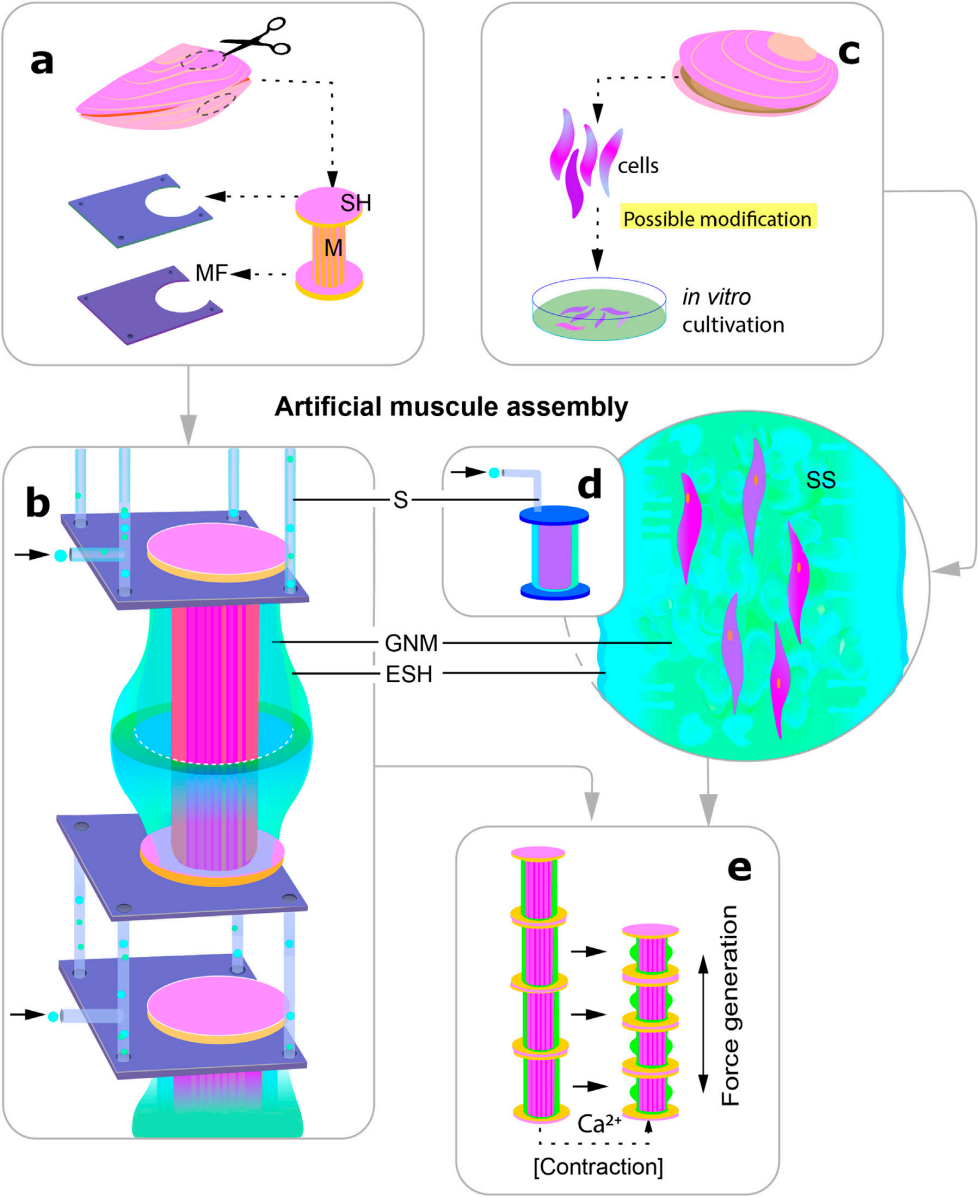
Scheme visualising the two possible ways of using catch muscles to create actuators in robotics. The different stages are indicated by the letters **(a–e)**. **(a)** extraction of the whole muscle together with segments of the shell for subsequent fixation in an artificial frame; **(b)** the muscle is surrounded by an artificial elastic shell in which gas exchange and nutrient media are supplied, individual segments are connected by frames through which media exchange takes place; **(c)** alternative way of creation: (genetic modification is possible); **(d)** transfer of the cells into the structural framework enclosed in a shell similar to that shown under **(b)**; **(e)** artificial muscle contraction: release of calcium into the medium leads to muscle shortening and bending of the elastic shell. M, muscle; SH, shell; MF, metal frame; S, channels for media supply to the muscle; SS, synthetic scaffold; GNM, gas exchange and nutrient medium; ESH, elastic shell.

If in the sections above we considered the structure and principle of operation of the catch muscles at the molecular and tissue level, now we would like to talk about the formation of muscles, their development in the body. This aspect may be useful if the creation of artificial muscles is done by growing muscles from cell culture rather than extracting muscles from a specially grown organism (Figure 7).

### 2.1 Myogenesis *in vitro*

With the development of cellular technologies, it has become possible to obtain individual muscle cells in cell culture conditions. Thanks to additional additives to the culture medium (transferrin, inulin), as well as neurotransmitters (acetylcholine and serotonin), it was possible to obtain myogenic differentiation of non-sterilized cells and even their reduction *in vitro*.

The embryonic and larval material of invertebrates is currently the most promising cellular material for obtaining primary cultures of invertebrates (Rinkevich, 1999; Rinkevich, 2005). The larval cells of trochophores and veliger bivalves still have sufficient proliferative potential for cell cultivation *in vitro* (Odintsova and Khomenko, 1991; Odintsova et al., 2010). However, the duration of such primary cell cultures does not exceed 1-2 month maximum (Odintsova et al., 2010).

**FIGURE 8:**
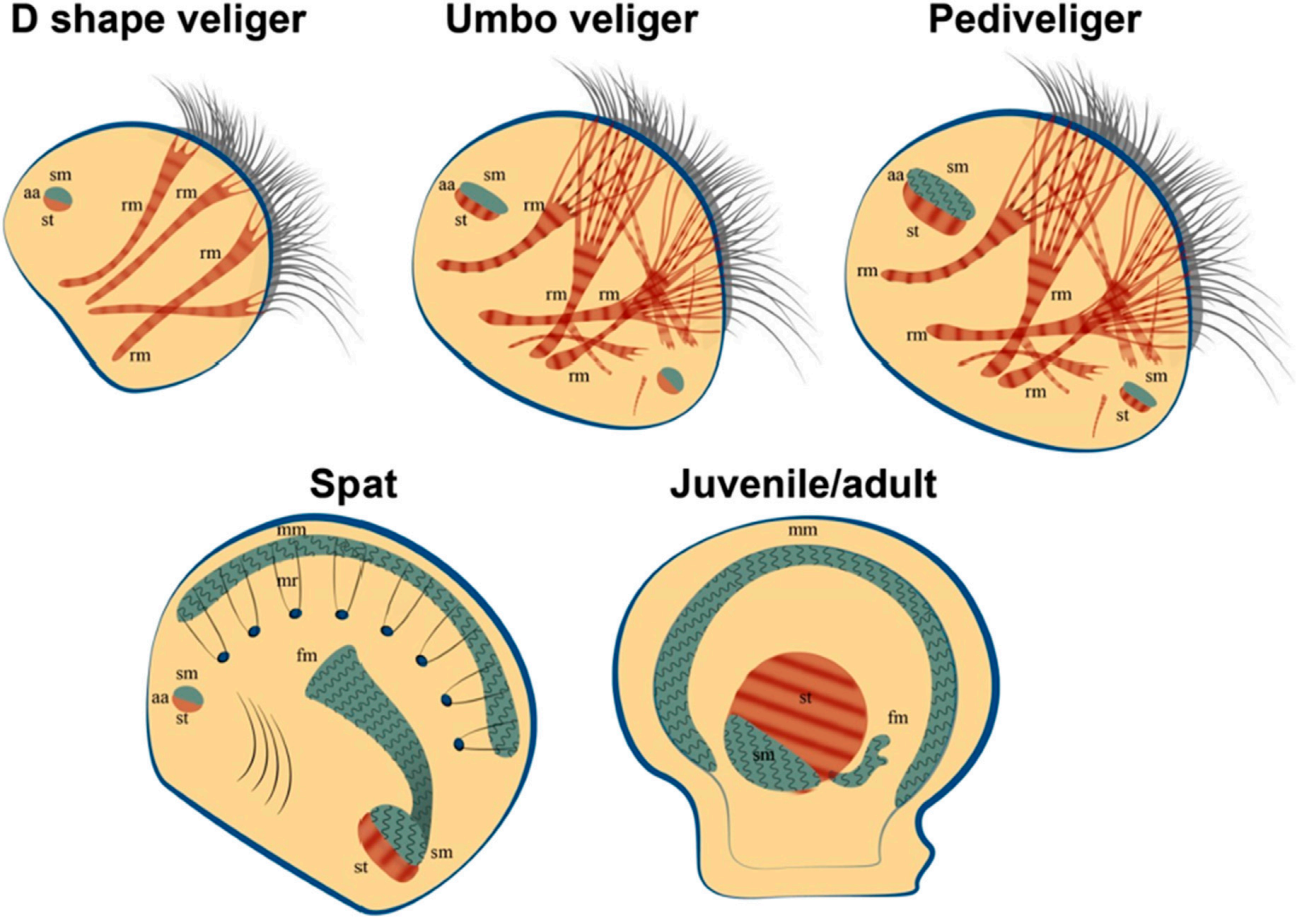
Bivalve myogenesis *in vivo*. The appearance of smooth muscle and its transformation in the later stages of development. Formation and growth of catch muscles in juvenile individuals. Abbreviatures: aa, adductor muscle; fm, foot muscle; mm, mantle muscle; mr, mantle retractor; rm, retractor muscle; sm, smooth muscle; striated muscle.

It has been shown that cells obtained from trochophores of the premyogenic stage of development (24 h of development) are able to reproduce (Figure 8) the process of organization of mollusc myofibrils *in vivo* (Odintsova et al., 2010; Dyachuk, 2013). The timing of myogenesis *in vitro* was surprisingly similar to those *in vivo*. In the culture of cells obtained from 24-h trochophores, muscle proteins appeared after 6 h of cultivation. Similarly, *in vivo* proteins were first detected at 30 hpf (Odintsova et al., 2010; Dyachuk, 2013). The striated pattern appeared after 36 hpf *in vivo* and 12 h in culture and was replaced by an even arrangement after 30 days *in vivo* (Odintsova et al., 2010; Dyachuk, 2013) and 20 days in culture. Actin was detected as one of the first detectable proteins in cells, which confirms its potential role in the initiation of myofibrillogenesis (Odintsova et al., 2010). Later, proteins of thick filaments (myosin, paramyosin, twitchin) appear, gathering into myofibrils with a striated pattern. Interestingly, the process of inhibition of myofibrillogenesis is reversible. Cells cultured on collagen (I type) do not differentiate into myocytes, but partially express muscle proteins (Dyachuk, 2013). After transferring these cells to fibronectin or glass coated with amorphous carbon, muscle cells and a clear pattern of striated myofibrils appear (Dyachuk, 2013). Surprisingly, after a day, some bipolar cells showed spontaneous rhythmic contractions. The number of contractile cells gradually increased during cultivation (but never exceeded 20% of the total number of cells). After 2 weeks of cultivation, the cell aggregates formed multilayered cell sheets, which were connected to each other by long spindle-shaped cells and also contracted. At the later stages of cultivation (more than 2.5 months, see additional material), a reduction of entire cell layers was observed (Odintsova et al., 2010). Of course, the limited life span of the described cultures, which is much shorter than that of the mollusc, makes the practical application of cell cultures of invertebrate muscles impossible for the time being. However, understanding of the mechanisms inducing cell differentiation and obtaining functional, contractile myocytes make this direction extremely promising for the production of synthetic muscle tissues.

### 2.2 Myogenesis: *in vivo* studies

While the biochemical and physiological aspects of muscle regulation of contraction in adult molluscs are well studied, little is known about the myogenesis of larval muscles (Dyachuk et al., 2005; Dyachuk and Odintsova, 2009). Bivalve molluscs generally have either one (in centre of body) or two (anterior and posterior) adductor muscles. Some species of bivalves have rarely even three adductor muscles (Huber, 2010).

**FIGURE 9:**
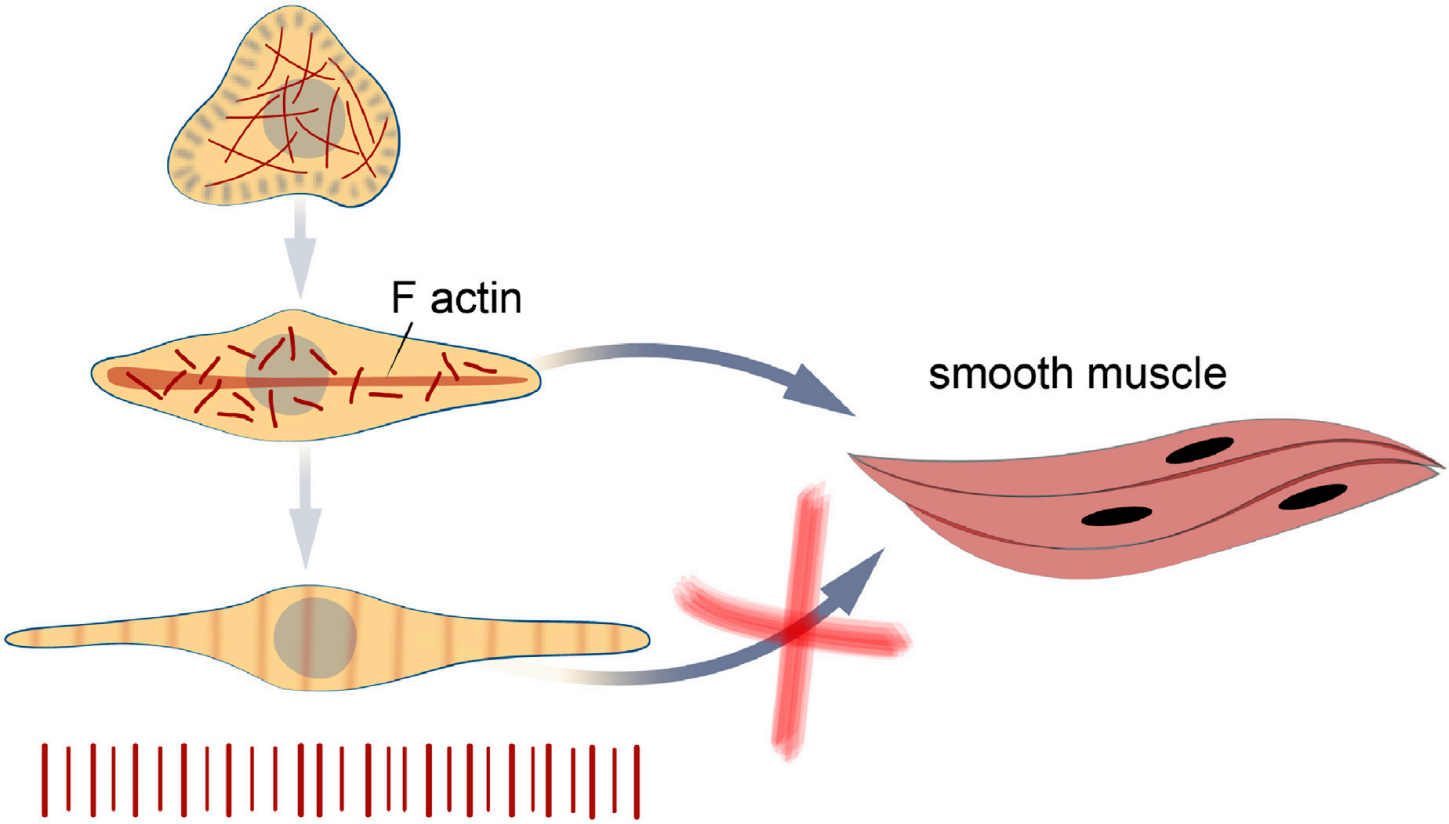
Bivalve myogenesis *in vitro.* The cells obtained at the trochophore stage are able to differentiate in two directions - into differentiation into striated and smooth myocytes. The transformation of striated muscles into smooth ones does not occur in cell culture, as it was found in larvae at late stages of development.

How is the muscular system of the larvae of bivalves formed? How are the smooth muscle and striated muscles of the larva formed and how are these processes related to the lifestyle and type of movement of larvae in the water column? All these questions can be answered by knowing when and how muscles are formed in larvae. It should be mentioned that bivalves have a non-direct development and a two-phase (plankton-benthic) life cycle. Thus, a blastula covered with cilia is formed from a fertilized egg, thanks to which it hovers (passive movement) and moves in the water column (not muscle movement). The blastula turns into a trochophore larva, which is characterized by the appearance of a shell germ and the beginning of the expression of muscular proteins (Dyachuk and Odintsova, 2009; Kas’yanov, 1989; Wanninger et al., 2016) and the appearance of the first non-functional muscle structures, which, as the trochophore develops into the next stage of development, veliger, turn into an anterior smooth muscle adductor (catch muscle) and 4-5 striated retractors (Figure 9). The veliger adductor already regulates the collapse and opening of the shells, and the retractors control a special ciliated ciliary organ - the vellum, which is involved in the capture of food and the movement of the larva’s body (Dyachuk and Odintsova, 2009).

It has been shown that smooth and striated muscles appear at the veliger stage of development in mussels, oysters and scallops. These data were obtained both by indirect immunofluorescence (using specially derived primary antibodies) and by the physical method of secondary harmonic generation (Dyachuk and Odintsova, 2009; Audino et al., 2015; Sun et al., 2019).

At later stages of larval development in a relaxed state, it is clearly possible to determine the type of larval muscles and the organ that they contract/ relax (for retractors). After about a month (in different species in different ways), the veliger turns into a pediveliger larva (Kas’yanov, 1989; Dyachuk et al., 2012; Cragg and Taylor, 1996; Malakhov and Medvedeva, 1985).

This stage of development undergoes a metamorphosis, which also captures the muscular system and turns into a juvenile individual of a full-grown mollusc. Pediveliger undergoes some changes: due to the resorption of vellum, velum retractors disappear, in oysters due to foot resorption, foot retractors disappear, the anterior adductor decreases in size (Yurchenko et al., 2018). At the same time, the rudiment of the posterior adductor, the mantle and gill muscles, consisting of thin microfibrils, are strongly developing (Yurchenko et al., 2018). It is noteworthy that at a fairly early stage of development, the mollusc already acquires a fully functional catch muscle, passing through metamorphosis. This opens the way to the study of miniaturised catch muscle and its application in micro robotics.

### 2.3 Neuromuscular development and transmitters regulation of catch

Understanding how a muscle is innervated is essential for developing artificial ways to stimulate synthetic muscles. Therefore, in this section of the review, we briefly reviewed the innervation of the molluscan catch muscle and its development.

The central nervous system of scallops consists of three main ganglia: the cerebral and pedal ganglia (CPG) and the visceral ganglion (VG). The VG of scallops is the largest and most complex ganglion found in bivalves, which is a complex neural data processing centre (Yurchenko et al., 2018; Kotsyuba and Dyachuk, 2022; Kniazkina and Dyachuk, 2022). The neurons that innervate the catch muscle are located in this ganglion. According to the classical catch regulation model, cholinergic nerves (producing acetylcholine – ACh) perform an excitatory function, while signals coming from serotonergic nerves (producing serotonin – 5-HT) lead to a rapid weakening of the grip force (Johnson and Twarog, 1960; Twarog, 1960). However, it has been shown that the catch muscles of larvae are innervated by serotonergic and FMRFamidergic neurons at the late stages of development of the mussel *M. trossulus* and the oyster *C. gigas* (Dyachuk et al., 2012). FMRFamide peptides have been shown to be key regulators of numerous central and peripheral functions in molluscs, such as interneuronal interactions (Kniazkina and Dyachuk, 2022; Too and Croll, 1995; Kotsyuba et al., 2020).

**FIGURE 10:**
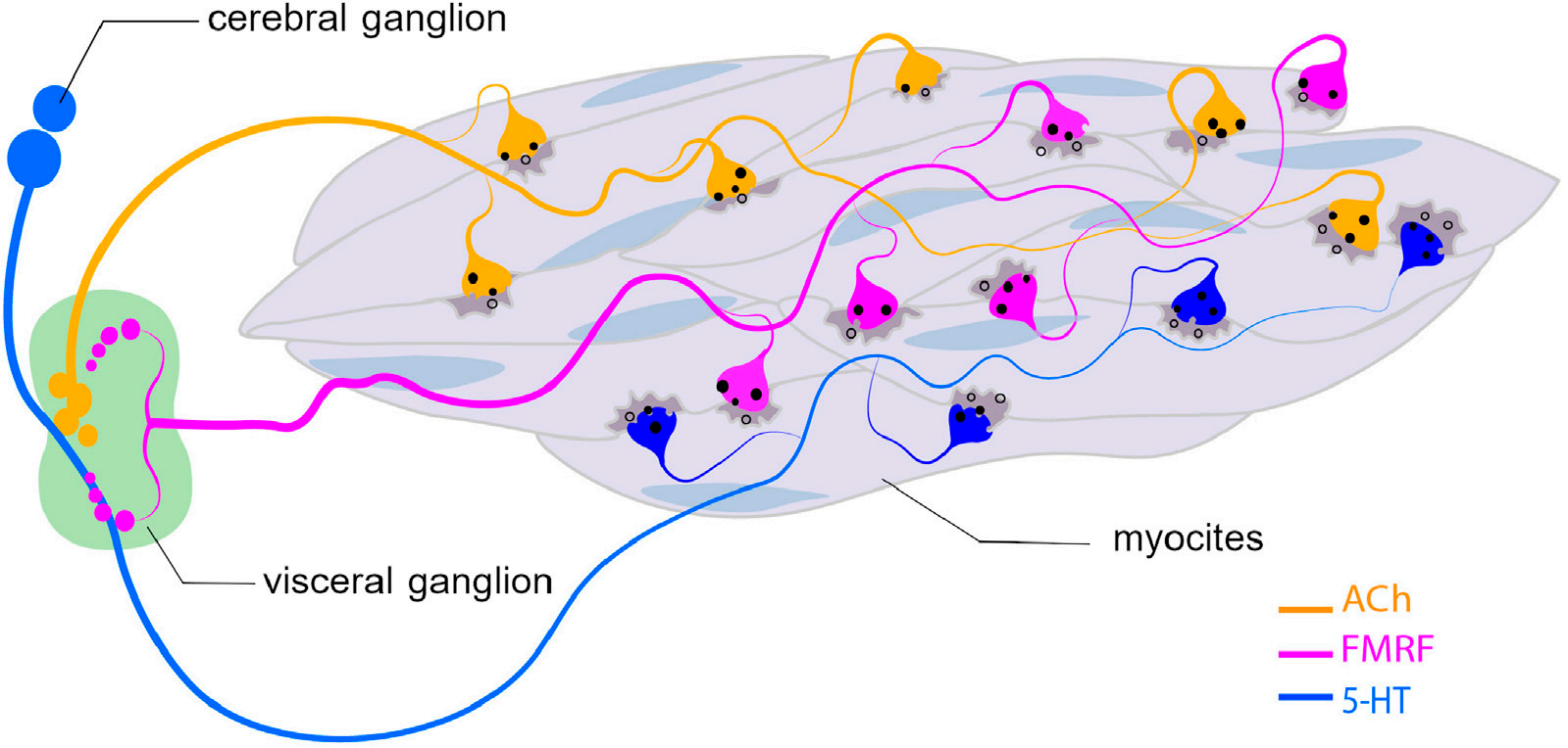
Innervation of catch muscle cells with ACh-, 5-HT-, and FMRMamydic neurons. Abbreviatures: ACh, acetylcholine; 5-HT, serotonin.

**FIGURE 11:**
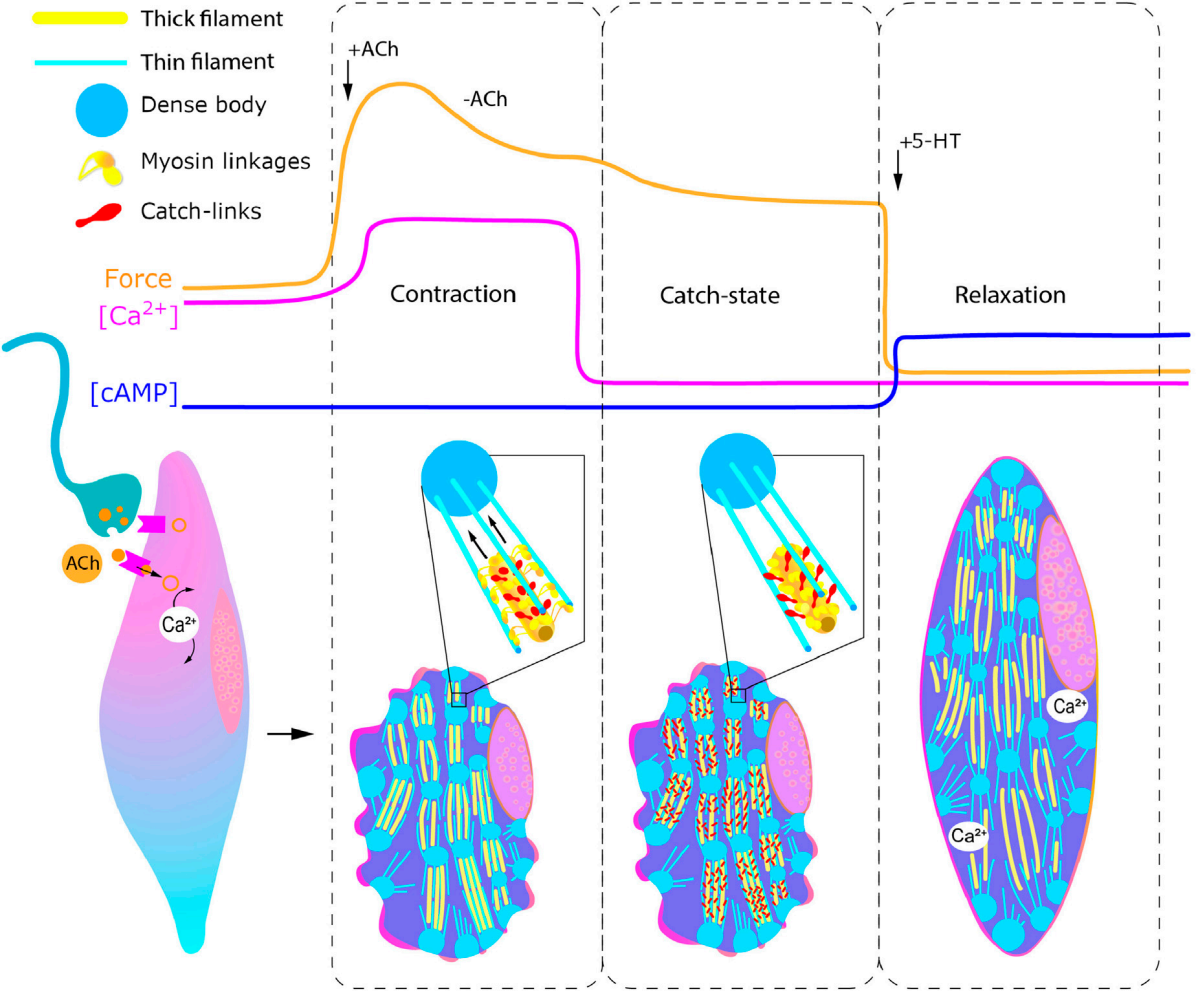
Regulation of catch muscle cell contractile states. Curves represent changes in force generation, calcium and cAMP concentration in response to acetylcholine and serotonin injection. Inset: enlarged representation of states of myosin linkages and catch links during contraction (left) and catch (right). Abbreviatures: ACh, acetylcholine; 5-HT, serotonin.

It was found that in both FMRFamidergic species, the fibers of paired pedal ganglia innervate the external adductor of pediveligers of both species. FMRFamidergic processes of visceral ganglia innervate the posterior adductor of the *C. gigas* oyster larva. The morphology of the terminal part of neurites, which innervate the anterior muscles, provides synaptic contacts for both FMRFamide and 5HT processes with smooth muscle (Dyachuk et al., 2012). In adult animals, such muscles are innervated by cholinergic and serotonergic nerves (Ishii et al., 1989). An integral scheme of catch muscle innervation is shown in Figure 10. Let us take a closer look at the process of neuroregulation of the catch muscle (Figure 11). When the catch muscles are stimulated by ACh, this leads to an increase in intracellular Ca^2+^ concentration, followed by a contraction and relatively high energy consumption and force production. After the stimulation stops, the muscle fibers relax very slowly or do not even relax completely. This state of slowly decreasing strength in the absence of stimulation is a catch state. During catch, energy consumption is low, and the intracellular concentration of free Ca^2+^ is similar to that at rest (Ishii et al., 1989). The catch force is weakened by the action of 5HT, which is released from the synapses of certain neurons (Twarog, 1960).

Serotonin induces an increase in intracellular cAMP (Achazi et al., 1974) and this activates protein kinase A, which, in turn, phosphorylates the muscle protein twitchin (Siegman et al., 1998; Butler et al., 2001), which leads to the termination of catch (Siegman et al., 1997; Siegman et al., 1998).

Thus, specific tonic and phase motor neurons can regulate various states of muscle activity (contraction, catch and relaxation). Moreover, the “catch” state may be the result of the activity of excitatory and inhibitory nerve fibres, and the latter sharply limit the duration of the reduced state of the muscle. Nevertheless, to date, it is impossible to give a clear answer to the question whether the condition of the catch is caused by muscle innervation a multitude of neurotransmitters in adult bivalves.

### 2.4 Neurons-muscle interaction components

scRNA-seq analysis revealed the presence of neurons and synapses in the scallop adductor. For example, NeuN is a neuron-specific nuclear protein that serves as a marker of neuronal maturation (Sun et al., 2021), as well as for vertebrates (Dyachuk et al., 2014).

It has been confirmed that NeuN is specific for neurons located in muscles. Moreover, we assume that these neurons may be necessary for the growth and survival of myocytes in the scallop muscles. A close relationship between muscles and neurons is also observed in other invertebrate species, such as *Drosophila* and *Aplysia californica* (Seecof et al., 1972; Montgomery et al., 2002). Neurons are able to synthesize and secrete soluble factors necessary for cell proliferation and differentiation (Montgomery et al., 2002). In addition, neurons can stimulate the formation of contractile fibrils in the myogenic culture of the *Manduca sexta* moth (Luedeman and Levine, 1996). The organizational role of neurons in myogenesis is important to consider in future work on the development of smooth muscle cell cultures.

Immunodetection of the synaptic density marker (PSD95) and MAP2 in the adductor scallop muscle provides convincing evidence that neurons and muscle cells are able to establish functional synapses in the striated adductor muscle.

Interestingly, PSD-95 performs a physical interaction between transmembrane synaptic protein NMDA receptors and NOS (Christopherson et al., 1999). NOS has been shown to successfully interact with PSD-95 (Jiang et al., 2016), and sequence analysis of the NMDA receptors of the oyster *Crassostrea. gigas* revealed PDZ domains (Vogeler et al., 2021). An expanded network of cells expressing the NMDA receptor from the base of the foot, including the glands, to the foot itself (Vogeler et al., 2021; Vogeler et al., 2020) may participate in the transmission of internal signals or responses to external environmental signals coming from the tip to the base of the foot.

PSDs are critically important for understanding the molecular basis of synaptic transmission and plasticity in animals (Sheng and Kim, 2011). Comparing the PSD proteins in molluscs and vertebrates, it becomes obvious that in vertebrates most of the scaffold proteins have more isoforms than in molluscs. However, there are families of scaffold proteins that are present in molluscs and other protozoa, but absent in vertebrates. Moreover, alternative transcripts of a single gene in invertebrates may contribute to an increase in the diversity of synaptic plasticity (Orvis et al., 2022). PSD95 immunodetection is evidence that neurons and muscle cells are capable of creating functional synapses in the striated adductor sinus muscle. The presence of PSD95 in cells suggests that this protein can affect neuromuscular junctions and stabilize their organization, as shown in other invertebrates (Chen et al., 2011). The results obtained prove that PSD95 can organize the molecular architecture of postsynaptic density proteins to maintain the functional properties of the neuromuscular system of invertebrates. Since PSD95 is known to play an important role in synaptic plasticity, it is assumed that the nervous systems of vertebrates and invertebrates may share common structural features of synapse organization and mechanisms of synaptic plasticity (Glanzman, 2010).

In bivalves, data on the synaptic connection of cells are fragmentary. Nevertheless, chemical synapses in the clam *Mactra sulcatoria* and *ark clam Anadara broughtoni* have been described in the ganglia neuropile. All the main types of classical chemical synapses are found in the visceral ganglia of molluscs (axoaxonal, axodendritic, axoship synapses) In the studied animals, axoship synapses were also found, formed by invagination of a dendrite outgrowth into a synaptic bud. There is a mixed population of synaptic vesicles in the axon near the presynaptic membrane. Such morphological diversity of synapses, described in bivalves, indicates a high level of plasticity of their neurons, as well as the diversity and possibilities of responses to external and internal signals (Kotsyuba and Kotsyuba, 2002).

## 3 Catch muscles by single cell transcriptomic analysis

So far in this work we have considered the physiology and molecular structure of smooth muscle cells of the catch muscles. However, when embarking on such a difficult task as creating artificial muscles, it is important to realise that no tissue consists of a single type of cells, even if they perform a key function of the tissue. In this section, we have compiled data on those cell types that have been found in the muscle tissues of bivalves.

With the development of omics technologies, it has become possible to identify and characterize cell populations of different tissues and their origin in both vertebrates and invertebrates. Sequencing of the transcriptome provides static images of gene expression in the adductor muscles of the scallop (Sun et al., 2021; Sun et al., 2018).

However, the diversity of cells and their regional differences prevent the possibility of comprehensively characterizing the subtypes of cell populations in the adductor muscles of the scallop. Recent advances in RNA sequencing at the level of individual cells (scRNA-seq) allow us to characterize gene expression in individual cells, shedding light on various cell types and their differentiated manifestations (Kolodziejczyk et al., 2015).

In addition, scRNA-seq studies also reveal new molecular markers and molecular features for each cell type. As shown for young *Yeso scallops*, only 11,583 cells were identified in the striated adductor muscle sample, whereas only 3,894 cells were found in the smooth adductor muscle sample. 20 different cell subpopulations of striated muscle were identified, while only 14 cell clusters were identified in smooth muscle. It should be noted that, unlike the striated part of the adductor, such cellular types as slow motion nerves, striated muscle epithelial cells, granulocytes, and macrophage-like cells are not found in the smooth part of the adductor in Yeso scallops. As it was revealed, a significant variety of cell types was observed in striated muscle compared to smooth muscle. All 20 cell subpopulations were found in striated muscle, whereas six cell clusters were absent in smooth muscle according to scRNA-seq data. Differentially expressed genes have been found that represent different molecular features in these cell clusters. The identified 20 cell subpopulations included smooth muscle precursor cells (SMPC), fast motor nerves (FMN), parietovisceral ganglion (PVG), mesenchymal stem cells (MSC), striated muscle precursor cells (STPC), smooth muscle epithelial cells (SMEC), glial cells (GC), slow motor nerves (SMN), striated muscle epithelial cells (STEC), pallial nerves (PN), cerebro-visceral connective (CVC), hyalinocytes (HC), Blast-like cells (BLC), osphradial ganglion (OSG), myoadhesive epithelial cells (MYEC), hemolymph epithelial cells (HEC), fibrocytes (FC), rhogocytes (RHO), granulocytes (GRC), and macrophage-like cells (MLC) (Sun et al., 2021). Thus, the cellular composition of the bivalve adductor muscle is a complex tissue system that includes non-muscular elements.

### 3.1 Glial cells

Among all cell types found within the catch muscle, glia cells are of particular importance after neurons and myocytes. Glial cells are one of the most important components of a complex nervous system. Scallop glial cells containing a dense packing of filaments are located between the two lateral lobes of visceral ganglion (Kotsyuba and Dyachuk, 2022). They function as a structural basis inside the ganglion (Spagnolia and Wilkens, 1983).

It has been shown that neurexin participates in the stabilization of neuron-glia interactions by forming adhesive septum junctions on cell membranes and promotes the growth of motor neurons involved in muscle contraction. For example, numerous transcripts of the neurexin gene (nrxn1) present in glial cells emphasize their functional role in neuron-glia interactions in the scallop nervous system (Hart and Hobert, 2018).

The stem-like function of glial cells is supported by the upregulation of the expression of the neural stem cell marker cdc42, which can maintain stem cells in a self-renewing state in adulthood by regulating the position of basal mitoses (Cappello et al., 2006).

Based on the analysis, it can be assumed that glial cells can perform a potential stem function in scallops. For example, the transcription factor tin performs an important function in the early mesodermal divisions of *Drosophila*, which is necessary for the development of the heart and somatic muscles (Bodmer, 1993).

Moreover, msp130 has been identified as a primary mesenchymal protein specific to the cell surface of marine invertebrates (Anstrom et al., 1987a). In the study, the coincidence of tin and msp130 in the GC cluster suggests that these conservative molecular regulators may be involved in the maintenance of stem cells and the determination of stem cell niches in both vertebrates and invertebrates (Anstrom et al., 1987b; Lam et al., 2006). As indicated, tin performs a crucial function in the specification or differentiation of mesoderm derivatives, which is usually found in a subset of the precursors of the heart and somatic muscles in *Drosophila* and *Caenorhabditis elegans* (Anstrom et al., 1987a; Lam et al., 2006).

The comparison makes it clear that there are numerous parallels in the molecules that help us understand the genetic basis of the specification or differentiation of mesoderm derivatives in both vertebrates and invertebrates. This suggests that tin and msp130 may serve as prototypes for searching for genes involved in the mesodermal division of vertebrates, the heart and somatic development.

Single cell data elucidate the complex biology of catch muscles at the level of individual cells. This provides valuable resources for marine invertebrate cell biology and effective interspecific comparisons of cellular and molecular components in vertebrates and invertebrates. The authors hope that the theoretical knowledge gathered here on the tissue structure, cell types, innervation, regulation, development and molecular mechanisms of the catch muscles will make it possible to reproduce it for human benefit.

### 3.2 Future application: artificial myofibrils, model system and nanorobots

This section is not intended to take the reader deeply into the technical problems of next-generation actuators, but seeks to summarise the present state of the problem. In this section we have tried to highlight the benefits that could be at the end of the road to the creation of artificial muscles, the foundations of which we laid in the previous sections.

Currently, all industry efforts are focused on the production of electric motors and internal combustion engines that convert the force of electric current and the combustion of petroleum products into mechanical work. The efficiency of such installations varies from 30% to 95%, but the total efficiency of the machines is much lower, and at best is 30% (Demirel, 2018; Dahham et al., 2022). This means that 2/3 of the energy expended by the engine is dissipated in the process of doing work. Losses of 2/3 of energy refer to losses solely by the engine itself; energy losses also occur during the delivery of fuel or electricity through power supply networks. According to rough estimates, on average they reach 15% (Sadovskaia et al., 2019) (in some cases even up to 25% (Bhatti et al., 2015)). The second aspect indicating the dead end of the modern branch of production of mechanisms for performing work is their low scalability. This, of course, is not about increasing the size of mechanisms, but about reducing classic electric and fuel engines to a microscopic scale. It is quite obvious that the mechanisms that outperform the developments currently used in industry lie right before our eyes - these are the structures underlying the motility and mobility of living systems. Such mechanisms have a number of advantages: a much longer service life (due to constant updating), greater efficiency, the ability to flexibly reduce and increase the scale of the mechanism that performs work from the microcosm to the macrocosm. All these advantages are fully recognized by the scientific and technical community. Therefore, biological systems have previously served and still serve as a unique source of inspiration when humans create and improve all kinds of machines. The history of aviation is a clear example. It begins with the myth of how birds’ feathers became the basis of the first aircraft and ends with the creation of modern, technically complex machines whose aerodynamic properties were undoubtedly copied from those of birds’ wings.

Progress is inevitable, and in the process of improving technology, one era of progress replaces another: the era of steam engines gave way to the era of electric ones. However, we are probably still a long way from fully understanding and routinely using biological mechanisms in industry. The main obstacle to the application of the principles of biological mobility in production is the lack of material and technical base for the creation, production, and operation of such bio-machines. The development of such a base must require the economic, environmental and social interests of humanity. At the moment, instead of such interest, there is more curiosity. Therefore, the examples of borrowing mechanisms (or principles of their operation) from biological systems considered in this section can only be quite conventionally called effective. Their full implementation requires the deepest study of application scenarios, adaptation of production means and, most importantly, serious refinement of the developments themselves. Despite this, we have tried to formulate a general idea of the possibilities of the next stage of technological progress and analyse emerging opportunities, accompanied by examples of both existing developments and only proposed technologies.

### 3.3 Types and examples of new generation actuators

The most significant examples of the development of artificial actuators inspired by the muscles of living beings can be divided into several groups: abiological actuators (dielectric elastomer actuators); bio-inspired mechanical actuators (e.g., drives operating by changing pressure); bio-actuators (derivatives of animal and plant tissues). Let’s take a look at these types of actuators and talk about their advantages and disadvantages.

**FIGURE 12:**
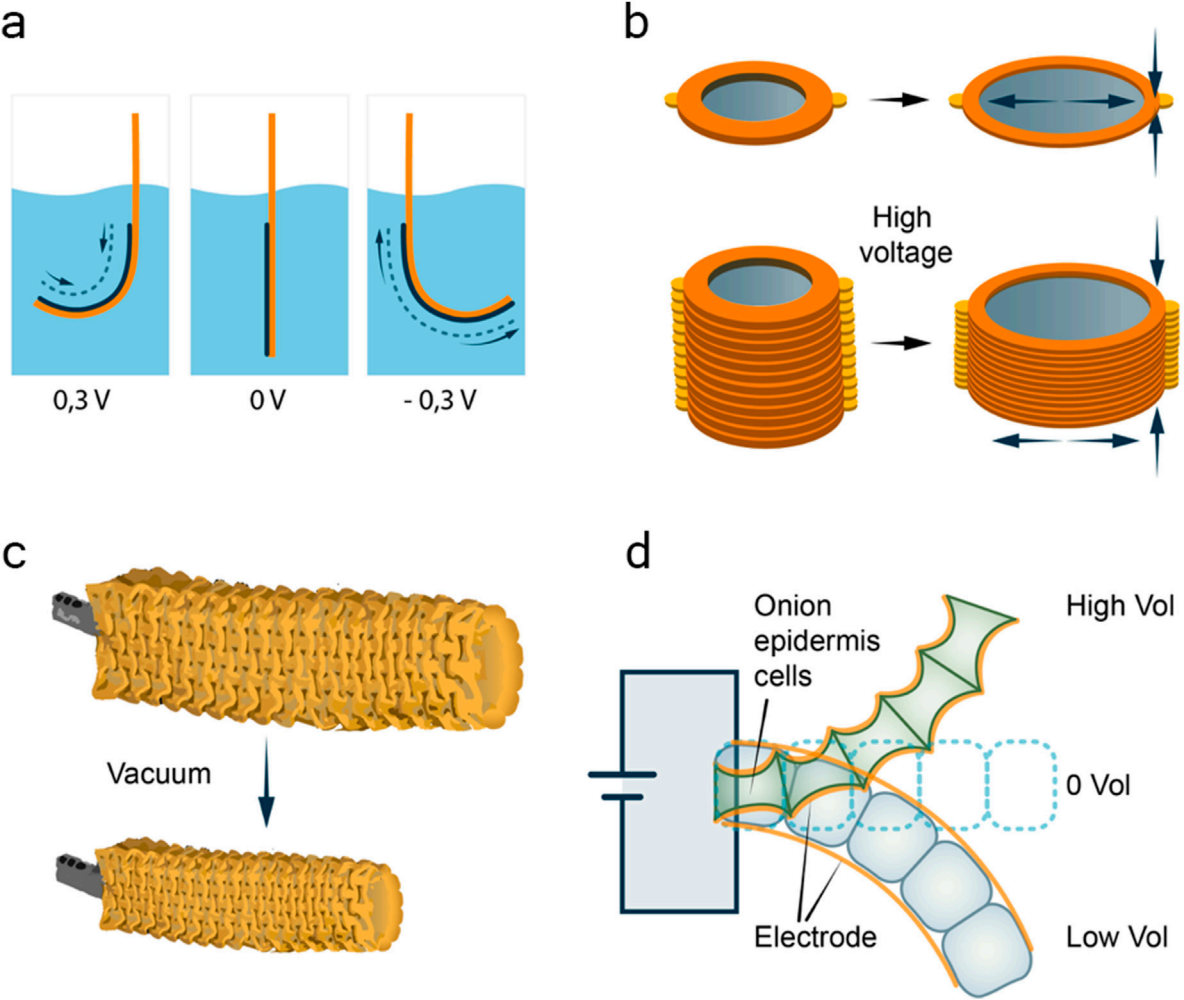
Types of artificial muscle-inspired actuators. **(a)** Scheme of electrochemical actuator, consisting of electrode films formed by restacking chemically exfoliated nanosheets of two-dimensional metallic molybdenum disulphide (black) on thin plastic substrates (orange). Medium is 0.5 M H_2_SO_4_. Based on (Acerce et al., 2017). **(b)** Scheme of dielectric elastomer actuator consisting of stacks of self-contracting active elements. Electrodes of carbon nanotubes are shown in grey, orange colour marks plates of acrylic elastomer. Based on (Qiu et al., 2019). **(c)** Work of soft pneumatic actuator. Actuation activated by negative pressure (vacuum); the tube on the left (grey) is connected to the vacuum pump. Based on (Yang et al., 2016). **(d)** Work of living tissue-based actuator from onion epidermal cells. Electrodes coating single layered onion cells marked with orange. Onion cells bending and stretching marked with blue and green, correspondingly. Based on.

The first of the considered types of actuators is abiological. The most suitable approach for designing micromotors, of course, seems to be one based on the use of electrically activated electrochemical actuators (Acerce et al., 2017) or dielectric elastomer (Qiu et al., 2019) (Figures 12a, b). In this case, mechanical work is performed due to a small change in the curvature, and, as a consequence, the length of the actuator when exposed to a weak electric current. Such technologies have been known for some time, and are gradually being improved to gain greater control flexibility and increase degrees of freedom, which were initially very small (Zhang et al., 2010). At their core, actuators of this type resemble sarcomeres of the skeletal muscles of vertebrates, and to perform complex actions requires a complex chain of such drives. The advantage of the technology is that such an elastomer does not require maintenance, has a huge service life (300,000 cycles (Duduta et al., 2019)) and can be very miniature. The main disadvantage is the inability to create macro-muscles capable of performing serious work, and dependence on the current source. Coupled with the problem of the impossibility of reducing the size of modern electric batteries without reducing their power (Fang, 2021), this technology acquires serious limitations in mobility. However, this type of actuators is quite suitable, with the proper level of modernisation, for solving tasks at the micro level such as - fine cleaning of mechanisms, control of micromanipulators (for scientific or even medical purposes), production of high-precision equipment, and others.

Another approach to creating artificial muscles associated with gas law (Figure 12c). This type of actuator can hardly be said to be related to biological systems, but it can be said that at its core it operates on similar principles. Therefore, we can call this type of actuator biologically inspired. Although it has been gaining popularity lately, it seems to us the least promising. With this type of actuator, the three-dimensional structure of the artificial muscle changes due to changes in gas pressure, similar to a balloon being filled with air (Yang et al., 2016). As a result, such muscles have both incredible advantages and serious disadvantages (Hines et al., 2017). On the one hand, they demonstrate incredible load capacity for their own weight (Durante et al., 2021). This is due to the use of a really very light polymer body. On the other hand, when calculating the load capacity of drives, the fact of using a classic vacuum pump as an “energy source” is ignored. It should be taken into account that: firstly, such a pump is obviously larger than the battery required to operate the elastomeric muscles (Wehner et al., 2014). Secondly, the vacuum pump itself obviously requires its own battery or power circuit. Both components have a very ordinary resource [especially in comparison with the polymer body, whose lifespan is very high: 1,000,000 cycles (Durante et al., 2021)] and service life. Moreover, the need to use such a pump calls into question the creation of truly tiny mechanisms (even comparable to dielectric elastomer ones), and limits their mobility. A good alternative solution for powering this type of drive is the use of heating elements built into the muscle structure (Miriyev et al., 2017). In this case, the pressure inside the system increases not due to the injection of gas, but due to the evaporation of the liquid contained in it, after heating the heater built into the body. In this case, the power supply is carried out, as in elastomeric drives, with a weak current. One of the unresolved disadvantages of this type of mechanism is its fragility. At the same time, the resulting parts in case of damage are indeed much safer than the fragments of classical electric motors and servos (Yang et al., 2016). As for the degrees of freedom of such muscles, due to the complex three-dimensional structure of the body, they are, of course, greater than those of the above-described dielectric elastomer actuators, but less than those of biological muscles (Wirekoh et al., 2019). It is this type of actuator that is most suitable for creating prosthetics, or robots that can perform some tasks involving contact with living beings. The reason for this is the soft, elastic surfaces and the safety in case of damage already mentioned above.

There is also a growing interest in biological actuators, which are actuators that directly engage living tissues. Good examples of the natural-biological approach to the implementation of artificial muscles are synthetic animal meat and actuators based on tissues isolated from living organisms. An example of the latter approach is the use of onion skins as a drive (Chen et al., 2015). This example is truly remarkable, since its implementation involves changing the shape of living tissue under the influence of a weak electric current (Figure 12d). This provides a bridge between the current approach to industrial mobility (electric and fuel engines) and biological mobility. However, as in the case of inorganic elastomers, the scalability of this approach cannot but raise questions. Obviously, in order to increase the productivity and absolute strength of such a “muscle,” onion tissue will have to be produced synthetically (the original fabric is quite small in size for industrial use). In addition, the service life of such an engine without maintaining the supply of nutrients to the tissue raises questions. On the other hand, plant tissues, despite not very efficient level of motor activity, are initially endowed with a semi-autonomous source of energy - their own solar batteries based on chlorophyll. Especially interesting is the possibility of using plant tissues in space industry, where autonomy and reproducibility (in which plants are hard to find equals) are more important than the speed of functioning. As a result, in our opinion, the complex application of plant tissue properties can solve the problems facing this area. Nevertheless, this path is probably as challenging as the reproduction of mollusc tissues. Synthetic meat, which is an artificial derivative of real animal tissue, is a completely different matter. The above problems are absent here. The scalability, controllability and performance of such drives seem to be very high (Seah et al., 2022). However, at the current stage, this kind of fabric, of course, serves more social than practical purposes. These synthetic muscles are not used for their intended purpose - as a biological motor, but as a substitute for animal meat for the needs of the food industry. In this way, it is intended to solve the economical, ethical and social problems of the exploitation of animals by humans (Rubio et al., 2020). It is worth emphasizing, however, that the approaches currently used to grow these muscle structures *in vitro* (Benny et al., 2022) may in the future be useful for the production of fully functional contractile structures for industrial use. The key here, in our opinion, is the ability to influence the size and shape of the produced material, as well as the ease of regulating its contractility - the same neurotransmitters can be used that are usually used in living systems. As for the shape and size of artificial muscles, it can be easily determined by limiting the growth of muscle tissue. However, an important nuance may be the choice of approach to muscle growth: there are two known strategies for engineering 3D skeletal muscle tissue. The first is a scaffold-based approach, in which cells are cultured in a three-dimensional fibrin-based matrix that serves to replicate the natural mechanical environment. The second is a scaffold-free approach. Scaffold-free approach involves seeding myogenic cells and fibroblasts under conditions that promote the formation of sufficient extracellular matrix for subsequent cell self-assembly into 3D tissue (Wang et al., 2019).As for the regulation of the resulting muscle tissue, of course, the type of transmitters used will depend on the type of precursor tissue used to produce the muscle. In the case of, for example, the production of an analogue of vertebrate skeletal muscles, acetylcholine innervation will be sufficient (Kuo and Ehrlich, 2015). To regulate the functioning of tissue based on smooth muscle cells, taken from, for example, bivalve molluscs (*Bivalvia*), at least a second type of innervation will be required in addition to acetylcholine (Galler et al., 2010) – serotonergic (Gies, 1986). Of course, the use of such developments requires a greater focus on solving related problems. For example, it is necessary to develop a shell for muscle tissue that protects it and does not interfere with work performance. Another problem is the creation of an artificial system for the delivery and distribution of a nutrient and gas exchange medium throughout the tissue. However, the huge advantage of this approach is that it is possible to use muscle cell cultures (Choi et al., 2021) in which the processes of contraction, recovery and energy metabolism are initially perfectly tuned. Therefore, the task of producing and using such muscles no longer seems impossible; all that is required is the development of the appropriate infrastructure. Among other things, it is worth pointing out the possibility of using modified muscle cells in technology, as well as the use not of tissues, but of individual synthetic contractile structures, or, for example, even independent proteins modified to solve specific problems. Thus, at the moment, the scientific community knows all the protein factors that control the synthesis of thin filaments, their attachment to membranes, branching and limiting their length (Lehman and Morgan, 2012; Ono, 2014; Szikora et al., 2022). The structure of thick filaments is also clear, the structural parameters of which can also be finely tuned (Ojima, 2019; Caremani et al., 2021). For example, regulate their size [length (Thoresen et al., 2013) and thickness (Levine et al., 1976b)], functional activity by varying the protein composition. In the last aspect, I would like to emphasize that, for example, myosins are now known with very different temperature stability, speed of operation, direct and indirect regulation of activation. Replacement of genes for certain thick filament proteins in muscle cells can allow fine regulation of the ability of such synthetic muscles to contract, fix the effort they have developed, and achieve relaxation. For example, the modification of muscle tissue to use specific myosin (fast ATP-hydrolysing vertebrate myosin or slow molluscan myosin, which has its own calcium sensitivity) and thus the creation of hybrid versions of muscles does not seem incredible. In addition, the use of individual myosin molecules or their polymers as a molecular motor that controls, for example, a molecular flagellum, may turn out to be a very promising solution for delivering drugs to targets inside the body. Especially considering the fact that such a machine can use not current or pressure as fuel, but ATP dissolved in the liquids of the target object (Gordon, 1986). We would especially like to note that it seems to us that the most promising within this direction is the use of smooth muscle cells, since each of them is a separate functional element of muscle tissue. This means that among other classes of artificial drives, this type has the widest scaling range: from one motor protein molecule to full-fledged muscles capable of performing work on a macroscopic scale.

### 3.4 Advantages of catch muscle-based actuators

The applicability of tissues and molecules originating from bivalve molluscs for the purposes described above deserves special attention. Some aspects and advantages of this approach have already been discussed in the sections above; here we would like to briefly summarise and draw new parallels. In contrast to the above-mentioned actuators of abiological origin (including bio-inspired ones), muscle tissue-based actuators can offer a number of advantages, each of which we will briefly describe below:

• Elasticity and softness – as organic tissues, bivalve muscles are of course safe, as they do not contain fragile parts or explosive/toxic substances such as lithium (found in batteries) or petroleum products. The risk of electric shock is also excluded.
• Environmental friendliness – this point in the question of bioactuators speaks for itself, although the use of the catch muscles here is no more justified than the muscles of any other biological object.
• Very high developed force (relative to mass) – obviously, structures designed by nature for one purpose - to move in space - do that quite well. But when we talk about the advantages of the catch muscle over, for example, skeletal muscle, which is used as the basis for the production of artificial animal meat - the difference in the force developed by the catch muscle is simply incredible.
• Tuning ability – since muscles of bivalves are very complex and contain many different muscle proteins and their isoforms, by regulating their expression, the properties of synthetic muscle can be changed to a significant extent. For example, by regulating the expression of paramyosin, it is possible to change the ratio of force to energy consumption of the muscle, and depending on the isoform of myosin expressed in the muscle, it is possible to influence the speed of the actuators based on it. Moreover, it may be quite realistic to create a hybrid myosin fibre containing myosins with different speeds activated by different pathways - either directly by calcium (smooth muscle myosin), by other proteins (vertebrate smooth muscle myosin), or by actin conformation (myosin of the striated muscles in the presence of calponin). The different ways in which myosin is activated in muscle allow for the creation of a molecular “transmission.” More of the individual benefits of catch muscle proteins are described in the relevant sections above.
• Extraordinary energy efficiency – in this paragraph, three advantages of the tissues of the catch muscles should be mentioned at once. Firstly, the general efficiency of this muscle. While molluscan myosin functions slowly and is therefore more energy efficient, these muscles are structurally capable of developing incredible force (see the first point). Second, the unique ability of the catch muscles – special state, which allows to maintain the developed force, stopping energy consumption. This aspect is particularly important when it comes to the choice of catch muscles as the basis for the creation of artificial actuators. The ability to suspend work without consuming resources or accumulating fatigue is an extremely useful option that distinguishes the mechanical actuators currently in widespread use. It is important to note that all muscle types are deprived of this ability, with the exception of the catch muscles. You can easily check this by trying to maintain a fixed posture for a while and assessing the effort required to do so. It is important to note that in order to put this ability of the catch muscles to practical use, it is not necessary to reproduce all their contractile structures, facing the difficulties described above (section 2.2). For this purpose, it is sufficient to transfer myorod or twitchin proteins and their corresponding kinases and phosphatases into already actively used vertebrate muscle cultures. As some authors have shown, invertebrate proteins can function correctly under these conditions (Avrova et al., 2009). The issue of transferring invertebrate genes into the vertebrate genome should not be a serious obstacle. The advantages of the state achieved in this case by the actuator - low energy consumption, arbitrary fixation - may well be useful both in industry (e.g., for cargo transfer) and in medicine (fixation of prostheses in certain states). Finally, the third aspect not discussed above is the energy exchange of molluscan tissues. Some molluscs are known to form symbiotic relationships with dinoflagellates, receiving part of their energy supply literally “from thin air” (Armstrong et al., 2018). In this sense, mollusc tissues could prove to be an incredibly interesting framework, capable of combining the photosynthetic capacity of plants and the active movement of animals. This aspect seems very promising to the authors, but its nuances are beyond the scope of this review.
• Scaling – this point takes into account the most obvious advantages of smooth muscles as the basis for the production of actuators. Molluscan muscles can fully function over an incredible range of size ranges, from a few micrometres (veliger catch muscle size), to tens of centimetres (Tridacna gigas adductor (Maynard and Burke, 1971)). Corresponding to such diversity, the spheres of application of these muscles and their derivatives can range from micromotors moving in the vessels of the body to actuators of bioorganic exoskeletons.
• Reparative potential and unlimited service life – this point is the most promising, and reflects the most outstanding advantage of any bioactuator over all other types of mechanism. However, the advantages that this pathway promises are proportional to the difficulties to be overcome. Nevertheless, it may well be that biosynthetic meat producers have shown the right way to obtain long-lived muscle cells culture. Indeed, in the process of producing a commercially viable product (synthetic meat), mechanisms and approaches could be developed that could be useful for basic research. It would be difficult to create them within the laboratory because of the incomparability of resources and the restrictions on their allocation. Given the specificity of tissues of marine organisms, which are delicacies, rich in vitamins, minerals, and of great nutritional value, such a way to create synthetic muscles may be quite successful. The existing maricultural practice in a number of countries confirms this (Chen et al., 2024).

## 4 Conclusions and future directions

Overall, here we review the nuances of functions, specific properties and myogenesis of catch muscle. Special attention has been paid to updating the knowledge of the structure of the contractile apparatus, because many findings have been made since its last full description. This information can be helpful for readdressing and taking a fresh look at the problem of catch state and its artificial implementation in biorobotics. Particular attention is paid here to the development of actuators of a biological nature, which are able to possess all the advantages of biological tissues: efficiency, scalability, unlimited potential for repair and a deep customisation. The authors hope that the presented review will not only draw the attention of researchers to the problem of the catch state, but also, will contribute to the movement of robotics along the indicated, extremely promising path.

## Glossary

EDTA: Ethylenediaminetetraacetic acid
Mn: myosin
MR: myorod
PM: paramyosin
TW: twitchin
A: actin
Tm: tropomyosin
Tn: troponin
CaP: calponin
CaM: calmodulin
HMM: Heavy meromyosin
LMM: light meromyosin
PEVK domain: domain found in titin and other proteins, consisting of mainly four amino acid residues: proline, glutamine, valine, and lysine
Asp: aspartic acid
Thr: threonine
Ser: serine
CAMK: Ca2+/calmodulin-dependent protein kinase
MLCK: Myosin light-chain kinase
PKA: protein kinase A
PP2B: Serine/threonine-protein phosphatase 2B
cAMP: Cyclic adenosine monophosphate
CPG: cerebral and pedal ganglia
VG: visceral ganglion
ACh: acetylcholine
5-HT: serotonin
scRNA-seq: Single‐cell RNA sequencing
psd95: postsynaptic density protein 95
MAP2: Microtubule-associated protein 2
GABA: gamma-aminobutyric acid
mGluR: metabotropic glutamate receptors
msp130: mesenchyme-specific protein 130
GC: glial cells
